# Structure-Based Analysis of Cefaclor Pharmacokinetic Diversity According to Human Peptide Transporter-1 Genetic Polymorphism

**DOI:** 10.3390/ijms25136880

**Published:** 2024-06-22

**Authors:** Ji-Hun Jang, Seung-Hyun Jeong

**Affiliations:** 1 College of Pharmacy, Sunchon National University, 255 Jungang-ro, Suncheon-si 57922, Republic of Korea; jangji0121@naver.com; 2 College of Pharmacy and Research Institute of Life and Pharmaceutical Sciences, Sunchon National University, Suncheon-si 57922, Republic of Korea

**Keywords:** PEPT1, *SLC15A1* exons 5 and 16, cefaclor, pharmacokinetics, inter-individual variability

## Abstract

Cefaclor is a substrate of human-peptide-transporter-1 (PEPT1), and the impact of inter-individual pharmacokinetic variation due to genetic polymorphisms of solute-carrier-family-15-member-1 (*SLC15A1*) has been a topic of great debate. The main objective of this study was to analyze and interpret cefaclor pharmacokinetic variations according to genetic polymorphisms in *SLC15A1* exons 5 and 16. The previous cefaclor bioequivalence results were integrated with additional *SLC15A1* exons 5 and 16 genotyping results. An analysis of the structure-based functional impact of *SLC15A1* exons 5 and 16 genetic polymorphisms was recently performed using a PEPT1 molecular modeling approach. In cefaclor pharmacokinetic analysis results according to *SLC15A1* exons 5 and 16 genetic polymorphisms, no significant differences were identified between genotype groups. Furthermore, in the population pharmacokinetic modeling, genetic polymorphisms in *SLC15A1* exons 5 and 16 were not established as effective covariates. PEPT1 molecular modeling results also confirmed that *SLC15A1* exons 5 and 16 genetic polymorphisms did not have a significant effect on substrate interaction with cefaclor and did not have a major effect in terms of structural stability. This was determined by comprehensively considering the insignificant change in energy values related to cefaclor docking due to point mutations in *SLC15A1* exons 5 and 16, the structural change in conformations confirmed to be less than 0.05 Å, and the relative stabilization of molecular dynamic simulation energy values. As a result, molecular structure-based analysis recently suggested that *SLC15A1* exons 5 and 16 genetic polymorphisms of PEPT1 were limited to being the main focus in interpreting the pharmacokinetic diversity of cefaclor.

## 1. Introduction

Cefaclor is a second-generation cephalosporin antibiotic and belongs to the beta-lactam family [1]. Cefaclor is known to inhibit bacterial cell wall synthesis by specifically binding to penicillin-binding proteins (PBPs) located within the bacterial cell wall [2]. As confirmed not only in in vivo clinical studies but also in vitro, cefaclor has excellent antibiotic effects against most strains of Gram-positive aerobes such as *Staphylococci* (including coagulase-positive, coagulase-negative, and penicillinase-producing strains), *Streptococcus pneumoniae*, and *Streptococcus pyogenes* (group A β-hemolytic *streptococci*) [3,4]. In addition, it is reported to have excellent antibiotic effects against Gram-negative aerobes such as *Escherichia coli*, *Haemophilus influenzae* (including β-lactamase-producing ampicillin-resistant strains), *Klebsiella pneumoniae*, and *Proteus mirabilis* [5]. Therefore, cefaclor is widely used clinically to treat bacterial diseases such as pneumonia and infections of the ear, lung, skin, throat, and urinary tract [6].

Past reports have suggested the importance of interpreting the inter-individual pharmacokinetic variation of cefaclor [7,8]. Even though the same formulation and dose of cefaclor are administered, significant differences in body exposure may occur between individuals, and this is closely related to the risk of treatment failure and resistance (due to the persistence of subtherapeutic drug concentrations in the blood) as well as the side effects (such as hypersensitivity, allergic reactions, gastrointestinal disturbances, and blood disorders) of cefaclor. Interpretation of cefaclor pharmacokinetic diversity between individuals will be of great help in strengthening the efficacy and safety information about cefaclor by enabling individualized pharmacotherapy based on scientific predictions, moving away from empirical clinical usage [9]. However, despite the frequent prescription of cefaclor and its global popularity, information regarding the interpretation of inter-individual cefaclor pharmacokinetic variation from a precision medicine perspective is still lacking. In particular, quantitative cefaclor pharmacokinetic diversity analysis and interpretation according to genetic factors have rarely been reported to date.

Cefaclor is a peptide analog that has been reported to be a substrate drug for human peptide transporter-1 (PEPT1) [10,11,12]. According to a past report [11], cefaclor was an interaction substrate with PEPT1 (inhibition constant of 10–14 mM) in cell monolayer uptake and transepithelial flux studies using the human colon carcinoma cell line Caco-2. Furthermore, it has been suggested that the transporter most importantly involved in the absorption of cefaclor into the systemic circulation is PEPT [12]. PEPT1 is expressed mainly in the apical membrane of the small intestine and is responsible for substrate absorption, while PEPT2 in the kidney regulates re-absorption from the primary urine [13]. Therefore, PEPT1 genetic polymorphisms have been a promising target covariate in interpreting the inter-individual pharmacokinetic variation of cefaclor, especially the absorption variation following oral exposure [7]. However, only the possibility was suggested, and no quantitative pharmacokinetic comparisons (even in vitro studies) or direct research reports on the effects of genetic polymorphisms were confirmed. This may be closely related to insufficient knowledge of PEPT1 structure and mechanism of action at detailed target points and difficulty in obtaining clinical data considering genetic factors. PEPT1 is a secondary active transport system mainly present in the intestinal tract and is involved in the absorption of di- and tri-peptides from the lumen to the blood stream [14,15]. The coding gene for PEPT1 is solute carrier family 15 member 1 (*SLC15A1*), and polymorphisms within the gene have been suggested to be closely related to the occurrence of inter-individual pharmacokinetic diversity of substrate drugs [16,17]. In particular, exons 5 and 16 have been considered key elements in interpreting the pharmacokinetic diversity of substrate drugs due to the frequent occurrence of mutations in the population and potential changes in PEPT1 structure and function through amino acid substitutions (as a non-synonymous point mutation) [17,18]. Since cefaclor is also a clear substrate of PEPT1, the possibility of explaining the variation in oral absorption of cefaclor based on genetic polymorphisms in exons 5 and 16 of *SLC15A1* has been continuously raised [7].

The main purpose of this study was to analyze the inter-individual pharmacokinetic diversity associated with the transporter of cefaclor, which had not been clearly identified previously, through in vivo pharmacogenomic analysis and molecular structure-based modeling approaches. In other words, we attempted to quantitatively analyze the degree of correlation between individual cefaclor pharmacokinetic diversity in *SLC15A1* exons 5 and 16 (in relation to the PEPT1 genetic element) at the in vivo level and interpret the related results based on molecular structure at the in-silico level. Through this study, it was expected that the understanding of the structure-function correlation of PEPT1 would be improved and largely resolve the question of whether cefaclor pharmacokinetic diversity in *SLC15A1* exons 5 and 16 can be interpreted. This study contributes to significantly reducing the gaps in existing knowledge regarding the interaction between cefaclor and PEPT1 and the structural effects of genetic polymorphisms and provides a useful perspective in interpreting the pharmacokinetic diversity of not only cefaclor but also PEPT1 substrate drugs.

## 2. Results and Discussion

### 2.1. Pharmacokinetic and Modeling-Based Covariate Analysis

**Figure 1:**
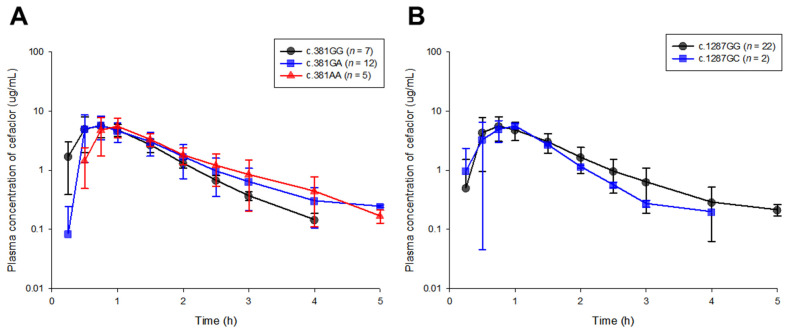
Comparison of cefaclor pharmacokinetic profiles (after oral administration of 250 mg) according to genetic polymorphisms of *SLC15A1* exons 5 (c.381G>A, (**A**)) and 16 (c.1287G>C, (**B**)). The dots in the graph represent the mean, and the vertical bars represent the standard deviation.

Comparisons of time-plasma concentration profiles between genetic polymorphism groups of *SLC15A1* exons 5 and 16 following a single oral administration of 250 mg of cefaclor capsule are shown in Figure 1.

Oral absorption of cefaclor began at 0.25 h after administration and rapidly progressed to approximately 1 h, and elimination from plasma occurred within 4–5 h after administration. Although plasma sampling was performed up to 6 h after oral exposure to cefaclor, cefaclor concentrations at 6 h were below the lower limit of quantification in all subjects. Interestingly, in the overall cefaclor pharmacokinetic profiles, there was relatively greater inter-individual variability in cefaclor plasma concentrations in the early absorption phase compared to the distribution and elimination phases. A past report had also noted absorption variability in the pharmacokinetics of cefaclor [7], and it had been suggested that analysis of the association of genetic polymorphisms in PEPT1 could be a key point in this regard. This was proposed based on previous reports that cefaclor was a clear substrate for PEPT1 [10,11]. Therefore, this study focused on newly exploring and interpreting the influence of genetic polymorphisms in *SLC15A1* exons 5 and 16 on the overall pharmacokinetics of cefaclor, including the absorption phase. This was because the effects of genetic polymorphisms in *SLC15A1* exons 5 and 16 on the inter-individual pharmacokinetic variability of cefaclor had not yet been clearly confirmed, and the results of pharmacogenomics research (related to *SLC15A1* exons 5 and 16) will be a very key element in the future precision medicine approach, not only for cefaclor but also for PEPT1 substrate drugs. A comparison of the pharmacokinetic profiles of cefaclor according to the genetic polymorphisms of *SLC15A1* exons 5 and 16 showed similar patterns between groups (Figure 1), and no statistical significance was identified at all plasma cefaclor concentration points (*p* > 0.05). Table 1 and Table 2 show the comparison results of pharmacokinetic parameter calculations between the genetic polymorphism groups of *SLC15A1* exon 5 and 16, respectively. Consistent with the results of the comparison of pharmacokinetic profiles, no statistical significance was identified between groups in the pharmacokinetic parameter values of cefaclor according to the genetic polymorphisms of *SLC15A1* exons 5 and 16 (*p* > 0.05). For the *SLC15A1* exon 5 genotypes in this clinical study population, the within-group proportions of c.381GG, c.381GA, and c.381AA were 29.17, 50.00, and 20.83%, respectively. In addition, for *SLC15A1* exon 16 genotypes, the within-group proportions of c.1287GG and c.1287GC were 91.67 and 8.33%, respectively, and the c.1287CC genotype was not identified. As a result, even though the number of subjects in this clinical study group was small (24 people), no large differences were identified from the results of PEPT1 genotype frequency analysis performed on a previously reported population of 519 Koreans (for *SLC15A1* exon 5 genotypes, the population proportions of c.381GG, c.381GA, and c.381AA were 30.4, 53.4, and 16.2%, respectively; for *SLC15A1* exon 16 genotypes, the population proportions of c.1287GG and c.1287GC were 88.8 and 10.0%, respectively) [17]. This indirectly implied that the results of the comparison of cefaclor pharmacokinetics according to the *SLC15A1* exon 5 and 16 genotypes performed in this study were satisfactory as a sample representative of the characteristics of the Korean population. The frequency of discovery of the *SLC15A1* exon 16 c.1287CC genotype in a large Korean population was only 1.2% [17], and the clinical data group used in this study was limited to 24 participants, so the c.1287CC genotype was inevitably not discovered.

The model equations that attempted to reflect the *SLC15A1* exon 5 and 16 genetic polymorphism elements as candidate covariates in the model are presented in Table S1. As a result of sequentially applying the information on genetic polymorphisms of *SLC15A1* exons 5 and 16 as categorical data to each parameter in the previously established and reported cefaclor population pharmacokinetic model structure [7], the genetic factors of *SLC15A1* exons 5 and 16 were not finally searched as effective covariates. In other words, compared to the existing model (as it did not reflect the *SLC15A1* genetic element), the model improvement according to the reflection of covariates did not reach a significant level (*p* > 0.05 and/or 0.01) in the quantitative numerical checks of twice the negative log likelihood (−2LL) and objective function value (OFV). In addition, the qualitative graphic check results showed that the goodness-of-fit (GOF) plots between the models before and after reflecting the *SLC15A1* genetic element were almost identical, and no notable points of improvement were identified. This suggested that the genetic polymorphism elements of *SLC15A1* exons 5 and 16 were not major factors with statistical validity in interpreting inter-individual cefaclor pharmacokinetic variation.

### 2.2. Molecular Modeling-Based Cefaclor-PEPT1 Interaction Analysis

**Figure 2:**
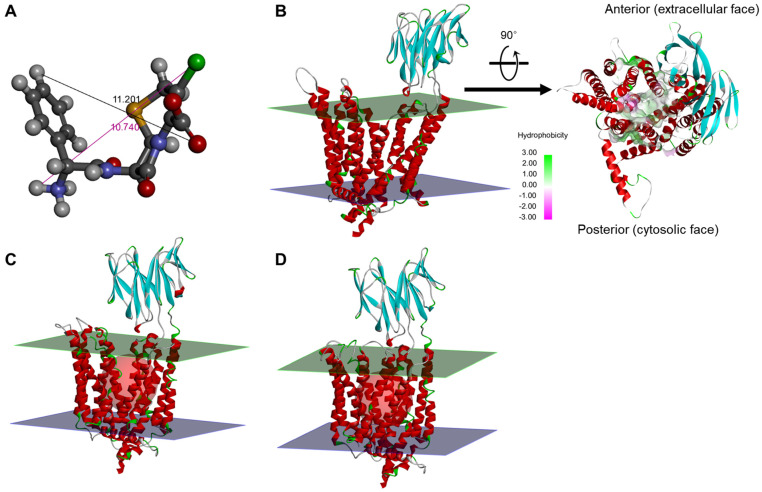
Three-dimensional structures prepared for cefaclor (**A**) and three outward-facing conformations (apo-state (**B**), open conformation (**C**), and occluded conformation (**D**)) of PEPT1. The lines and values shown in the cefaclor structure refer to the molecular lengths (Å) in the optimized structure at pH 7.4. Red spheres in PEPT1 outward-facing open and occluded conformations represent drug binding pockets discovered based on structural information and interaction energy with cefaclor. The green and blue planes in PEPT1 structures represent extracellular and cytosolic membranes, respectively. The additional arrow shown in (**B**) shows the solvent hydrophobicity surface analysis of the ligand entry site in the protein after rotating the structure so that the extracellular side faces forward in the PEPT1 outward-facing apo-state.

Since significant differences in cefaclor pharmacokinetics between groups according to genetic polymorphisms of *SLC15A1* exons 5 and 16 were not identified in clinical trial results-based research, an attempt was made to expand to PEPT1 and cefaclor structure-based modeling to perform molecular mechanistic interpretation. Figure 2 shows information on the three conformations of PEPT1 outward-facing, the three-dimensional conformation structure for the target substrate cefaclor, and the location of the drug binding pocket (DBP).

The created structures were optimized at 7.40, the typical body pH, and had an isoelectric point of 8.35 and a net charge of 5.04. In addition, sheet-shaped lobes were located in the extracellular direction along with 12 transmembrane helix aggregates, and a helical linker structure of amphipathic nature was arranged in the cytoplasmic direction. The linker was a bundle bridge that largely connected the transmembrane helix 1–6 (as an N-bundle) and 7–12 (as a C-bundle) assemblies. The molecular size of cefaclor, which was established as a ligand for PEPT1, was approximately 10–12 Å. Therefore, the space (radius) of DBP in PEPT1 open and occluded conformations was set to 13 Å, slightly larger than that of the ligand cefaclor. Also, based on the established DBP space, a search for specific domains that form strong and stable bonds with the ligand was performed. As a result of structural analysis of PEPT1’s outward-facing apo-state, the ligand entry site in the protein had a balanced distribution of hydrophobic and hydrophilic parts, but the degree of hydrophilicity in the deep part of DBP tended to be relatively high. This was interpreted to be related to the preferential environment for DBP binding within PEPT1 of cefaclor and other di- or tri-peptide substrates, which are hydrophilic. Figure S1 shows the results of analysis of the structural physicochemical properties of PEPT1 outward-facing apo-state. Analysis of the structural physicochemical properties of PEPT1 outward -acing apo-state showed that the surface hydrophobicity of the cell membrane penetrating regions was high and the interpolated and partial charges were close to 0. On the other hand, the surface hydrophilicity of the outer regions of the cell membrane was high, and the interpolated and partial charges had polarity values that deviated from zero. It was interpreted that the high surface hydrophobicity in the transmembrane regions is associated with the structural stabilization of PEPT1 as a transmembrane protein, and the surface polarity in the extracellular regions is associated with protein stabilization and matrix interactions in extra- and intracellular fluid. The Psi and Phi surface analysis results suggested that relatively α-helical amino acid residues were located in the transmembrane regions of PEPT1, while β-conformational amino acid residues were mainly distributed in the outer regions of the cell membrane. This was interpreted to be associated with the induction of the stabilization of PEPT1 within the cell membrane through strong binding with a large surface area and hydrophobic interactions. Figure S2 shows the Ramachandran plot results for the three conformations of PEPT1 outward-facing. The Ramachandran plot provides an easy way to view the distribution of torsion angles in a protein structure. It also provides an overview of allowed and disallowed regions of torsion angle values, serving as an important factor in the assessment of the quality of protein three-dimensional structures. The local backbones of all PEPT1 outward-facing conformations corresponded to the β-sheet and α-helix, and overall, no notable local backbone changes were identified between the apo-state and open and occluded conformations. This suggests that in the PEPT1 outward-facing state, three-dimensional structural changes due to ligand binding occur without significant structural torsion or secondary structure deformation of each local backbone, and that the three conformations of PEPT1 outward-facing generated in this study are acceptable structures without major overall problems.

**Figure 3:**
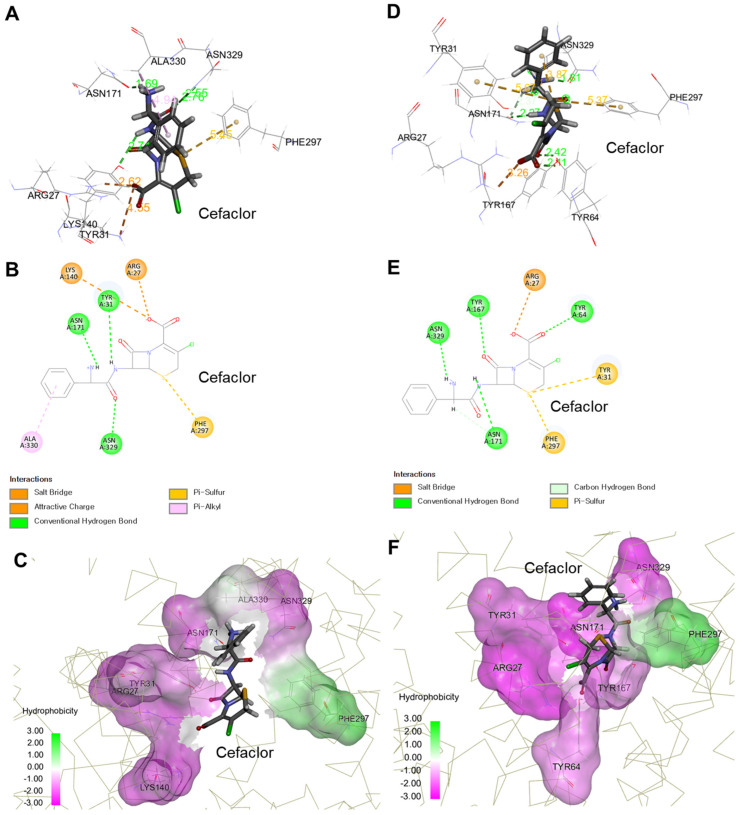
Simulation results of cefaclor docking within the drug binding pocket (DBP) in PEPT1 outward-facing open (**A**–**C**) and occluded (**D**–**F**) conformations. The colored dotted lines in the figures (**A**,**B**,**D**,**E**) indicate interactions between cefaclor and amino acid residues in PEPT1 DBP. (**A**,**D**) show a three-dimensional diagram of the receptor-ligand interaction, and (**B**,**E**) show a two-dimensional diagram of the receptor-ligand interaction. (**C**,**F**) show the results of solvent hydrophobicity surface analysis for receptor-ligand interaction (proteins are represented as gray-colored carbon α-wires).

The three- and two-dimensional interaction analyses and docking simulation results with cefaclor in DBP of PEPT1 outward-facing open and occluded conformations are presented in Figure 3.

The three-dimensional arrangement of cefaclor in DBP was that the carbonyl and carboxyl backbones were mainly arranged on the hydrophilic solvent surface, and the other sulfur and carbon aliphatic/aromatic backbones were arranged on the hydrophobic solvent surface. The –CDOCKER interaction energies following cefaclor docking to DBP in PEPT1 outward-facing open and occluded conformations were 52.97 and 53.78, respectively. The high –CDOCKER interaction energy values above 30 suggested that cefaclor corresponds to a very friendly substrate of PEPT1 and that the docking of cefaclor in DBP explored in this molecular modeling study was achieved with stable energy. The high –CDOCKER interaction energy values were interpreted to be due to non-covalent bonds including hydrogen bonds, salt bridges, and π-sulfur by 27 Arg, 31 Tyr, 171 Asn, 297 Phe, and 329 Asn in cefaclor and PEPT1 DBP. Additionally, it was confirmed that the total energy of −2257.04 kcal/mol in PEPT1 outward-facing apo-state was significantly lowered to −2870.22 and −3195.87 kcal/mol in open and occluded conformations, respectively. This was interpreted to be because cefaclor binds to the DBP of the PEPT1 outward-facing apo-state, forming a specific substrate interaction, resulting in stabilization of the overall protein structure. In addition, when PEPT1 changes from outward-facing open to occluded conformation, the addition of hydrogen bonds with cefaclor by 64 Tyr and 167 Tyr occurs, and the resulting enhancement of receptor-ligand interaction was interpreted as a factor accelerating the stabilization of the overall protein structure. As a result, considering the receptor-substrate interaction energy and structural stabilization patterns comprehensively, it was confirmed that cefaclor is a preferred substrate of PEPT1.

Figure S3 shows the results of the analysis of the operating mechanism through structural comparison between the three conformations of PEPT1 outward-facing. Qualitative comparison between each conformation was performed through alignment and superposition of the structure, and quantitative confirmation of the steric change was performed through calculation of the root mean square deviation (RMSD). It was confirmed that when changing from PEPT1 outward-facing apo-state to open conformation, large rotation of the N-bundle, small rotation of the C-bundle, and upward stretching of the bundle bridge appear. In addition, it was confirmed that when changing from PEPT1 outward-facing open conformation to occluded conformation, additional upward stretching of the bundle bridge appears along with minor rotation of the N-bundle and C-bundle. The degree of change from PEPT1 outward-facing apo-state to open conformation was relatively higher than the degree of change from open conformation to occluded conformation, and the RMSD values were 2.59 and 2.44 Å, respectively. The overall structural change of PEPT1 was that both the N-bundle and the C-bundle converged toward the central axis of the protein, which was interpreted to be related to the protein structure switching from outward-facing to inward facing as the ligand cefaclor binds to PEPT1 DBP. In other words, when cefaclor is transported from the outside to the inside of the cell, the outward-facing bundles of PEPT1 simultaneously turn inward, enabling unidirectional absorption of cefaclor from the outside to the inside.

### 2.3. PEPT1 Structural Analysis According to Genetic Polymorphisms of SLC15A1 Exons 5 and 16

**Figure 4:**
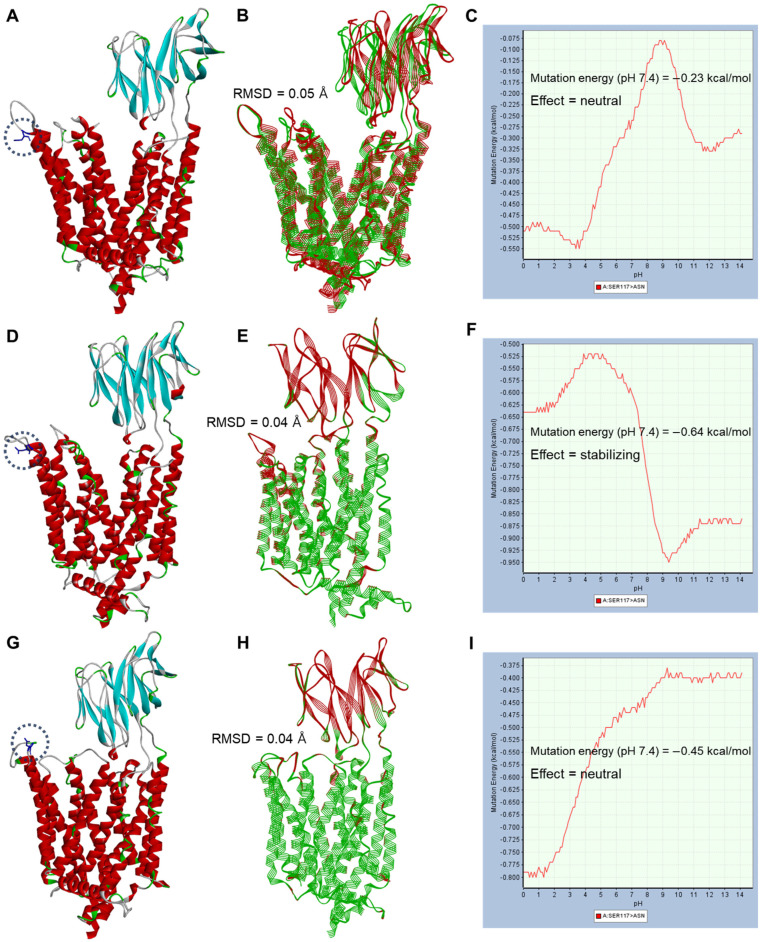
Structural analysis of amino acid substitution results (p.Ser117Asn) according to genetic polymorphism of *SLC15A1* exon 5 (c.381G>A) in three outward-facing conformations (apo-state (**A**–**C**), open conformation (**D**–**F**), and occluded conformation (**G**–**I**)) of PEPT1. Blue dotted lines in the structure indicate amino acid substitution sites in each conformation. Point-mutated amino acid residues are indicated as stick-type atoms. (**B**,**E**,**H**) show graphical comparisons and root mean square deviation (RMSD) results through alignment and superimposition between wild-type (green-colored line ribbon display style) and point mutation structures (red-colored line ribbon display style). (**C**,**F**,**I**) show the pH-dependent mutation energy (as stability) profile of PEPT1 following the point mutation. The mutation effects (**C**,**F**,**I**) were determined based on mutation energy values of ±0.50 kcal/mol.

**Figure 5:**
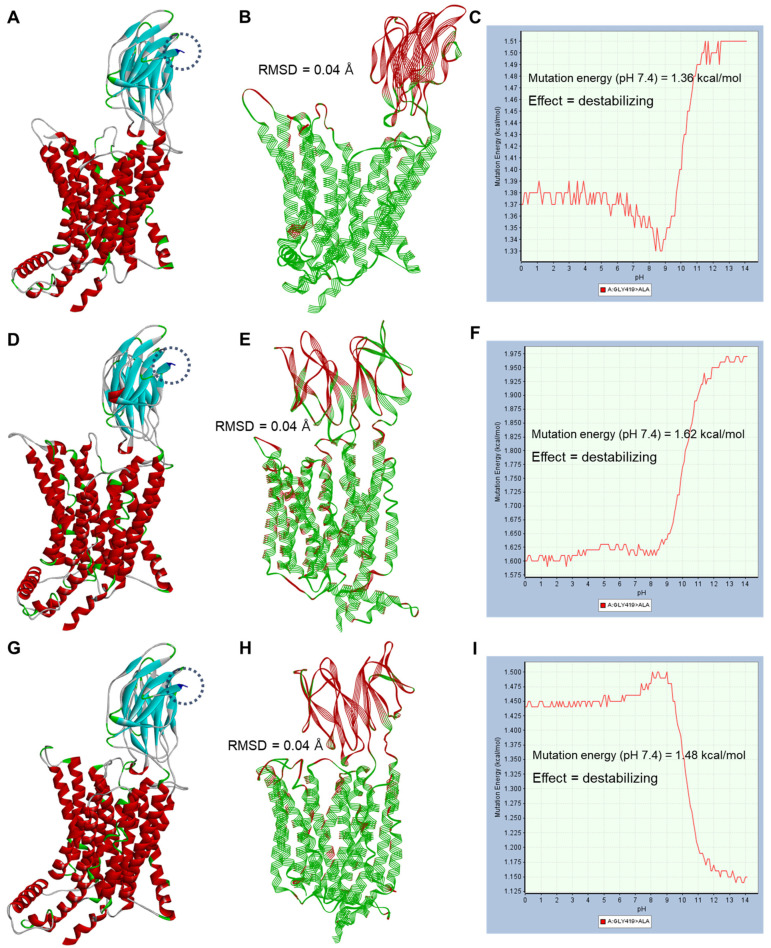
Structural analysis of amino acid substitution results (p.Gly419Ala) according to genetic polymorphism of *SLC15A1* exon 16 (c.1287G>C) in three outward-facing conformations (apo-state (**A**–**C**), open conformation (**D**–**F**), and occluded conformation (**G**–**I**)) of PEPT1. Blue dotted lines in the structure indicate amino acid substitution sites in each conformation. Point-mutated amino acid residues are indicated as stick type atoms. (**B**,**E**,**H**) show graphical comparisons and root mean square deviation (RMSD) results through alignment and superimposition between wild-type (green-colored line ribbon display style) and point mutation structures (red-colored line ribbon display style). (**C**,**F**,**I**) show the pH-dependent mutation energy (as stability) profile of PEPT1 following the point mutation. The mutation effects (**C**,**F**,**I**) were determined based on mutation energy values of ±0.50 kcal/mol.

Figure 4 and Figure 5 show the structural analysis of the amino acid substitution results as a point mutation (missense) according to the genetic polymorphisms of *SLC15A1* exons 5 and 16, respectively.

The amino acid substitution sites corresponded to the N-bundle (for exon 5) and C-bundle (for exon 16) of PEPT1 and were commonly located in sites physically distant from the DBP of PEPT1. Figure S4 shows the polymorphic positions of *SLC15A1* exons 5 and 16 in the topology diagram of PEPT1 and the positional information of key residues for interaction with cefaclor discovered in this study. As reaffirmed in the topology diagram of PEPT1, the polymorphic sites in *SLC15A1* exons 5 and 16 were physically distant from the positions of key residues interacting with cefaclor, and the interpretation of functional relevance was limited. In addition, in a comparison between wild-type and point mutation conformation structures through align and superimpose, the RMSD values were 0.04–0.05 Å, indicating that amino acid substitution did not cause a large three-dimensional structural change in the protein. Mutation energies (as stability) at pH 7.4 according to *SLC15A1* exon 5 genetic polymorphisms ranged from −0.23 to −0.64 kcal/mol, and the effects were neutral and stabilizing. The effectiveness judgments here were performed based on mutation energy values, and ±0.50 kcal/mol, which was set as default in BIOVIA Discovery Studio’s mutation protocol, was the reference point. In other words, if the mutation energy values were greater than 0.50 kcal/mol, the effect of the mutation was judged to be destabilizing; if it was less than −0.50 kcal/mol, it was judged to be stabilizing; and if it was within ±0.50 kcal/mol, it was judged to be neutral. The mutation energies (as stability) at pH 7.4 according to *SLC15A1* exon 16 genetic polymorphisms ranged from 1.36 to 1.62 kcal/mol, and the effects were all destabilizing. Figure S5 shows the structural analysis of the amino acid substitution results from concurrent genetic polymorphisms in *SLC15A1* exons 5 and 16. The mutation energies (as stability) at pH 7.4 according to the simultaneous genetic polymorphisms of *SLC15A1* exons 5 and 16 ranged from 0.78 to 1.31 kcal/mol, and the effects were all destabilizing. Mutation energy profiles according to pH changes in interaction with cefaclor according to genetic polymorphisms of *SLC15A1* exons 5 and 16 are presented in Figure S6. The mutation energies for cefaclor interaction at pH 7.4 ranged from 0.00 to 0.01 kcal/mol, and the effects were all neutral. As a result of the analysis of the PEPT1 structure and interaction with cefaclor according to the genetic polymorphisms of *SLC15A1* exon 5 and 16, it was suggested that the genetic polymorphism in *SLC15A1* exon 5 (c.381G>A) had little effect on the structural stability of PEPT1, but the genetic polymorphism in exon 16 (c.1287G>C) caused structural instability of PEPT1 relatively more than exon 5. However, considering that the instability value was not large at a maximum of 1.62 kcal/mol and the substrate interaction energy values due to mutation were close to 0.00 kcal/mol, it was suggested that the impact of *SLC15A1* exon 16 genetic polymorphisms on cefaclor interaction may be very negligible.

As an additional extended interpretation, it is predicted that if it is a beta-lactam drug with a similar structure to cefaclor and is a substrate for PEPT1, a phenomenon similar to the result of the cefaclor-PEPT1 interaction proposed in this study will result. In other words, the binding of substrate drugs within the DBP of PEPT1 derived from this study is located at a position that has no significant physical correlation with *SLC15A1* exons 5 and 16 and is not expected to have a major effect on transporter-substrate interactions. As a result, the effects of genetic polymorphisms in exons 5 and 16 of *SLC15A1* were not expected to be significantly related to the inter-individual pharmacokinetic diversity of cefaclor-like beta-lactam drugs that are PEPT1 substrates. A past report [11] suggested that, in addition to cefaclor, cefadroxil, cefamandole, cephradine, cefuroxime, cefixime, cephalotin, cephalexin, and ampicillin may have substrate interactions with PEPT1.

### 2.4. PEPT1 Structural Analysis through Application of Other SLC15A1 SNPs

Figure S7 shows the location information of elements related to amino acid substitution by missense as a result of the previously reported *SCL15A1* single nucleotide polymorphisms (SNPs) [18] in the three conformations of PEPT1 outward-facing. Furthermore, Table 3 shows the structural stabilization of PEPT1 and the results of the binding energy analysis with cefaclor as a result of applying the previously reported *SCL15A1* SNPs [18] in the three conformations of PEPT1 outward-facing. It was determined that the nine previously reported missense-causing SNPs in *SCL15A1* [18] had very minimal effects on cefaclor transport by PEPT1. This was because the degree of impact on PEPT1 structural stability was not significant (the maximum increase in stability energy value was 1.62 kcal/mol), and the binding energy changes with cefaclor were all less than 0.50 kcal/mol, meaning that the mutation effects were neutral. Figures S8 and S9 show the energy analysis profiles of the binding structure with cefaclor in PEPT1 outward-facing open and occluded conformations, respectively, based on the application of point mutations in the key residues of the interaction between cefaclor and PEPT1 discovered in this study. Table 4 summarizes the amino acid substitution results that showed the highest bond structure energy change (instability) in the energy analysis profiles in Figures S8 and S9. As a result, it was confirmed that the key residues for the interaction between cefaclor and PEPT1 discovered in this study are physically located within DBP, and that when replaced with other amino acids, they induce a significant increase in the binding energy change (more than 0.50 kcal/mol), causing binding instability (between cefaclor and PEPT1) in the overall effect of the mutation.

Figure S10 shows the results of the PEPT1 structural stability analysis profile through alanine mutagenesis in three conformations of PEPT1 outward-facing. The attempt at alanine mutagenesis was to screen for specific amino acids within the PEPT1 structure that affect protein stability and/or function. Alanine mutagenesis was performed by measuring changes in PEPT1 structural energy as each amino acid was sequentially substituted with Ala in the control PEPT1 amino acid sequence using a combinational amino acid scanning protocol. Table 5 shows information on the top five amino acids causing structural energetic instability in the PEPT1 structural stability analysis profiles through alanine mutagenesis for the three conformations of PEPT1’s outward-facing (Figure S10). Moreover, the steric positions of the corresponding amino acids in PEPT1 outward-facing apo-state and open and occluded conformations, as well as the interaction information with surrounding amino acids, are presented in Figure S11. Ala substitutions in the PEPT1 structures of amino acids screened by alanine mutagenesis all resulted in a structural instability effect of more than 0.50 kcal/mol, which suggests that these amino acids may be important residues involved in maintaining the operation and stability of PEPT1. In addition, when confirmed based on PEPT1 structural information, the amino acid positions screened by alanine mutagenesis were commonly hubs of multi-bond interactions based on strong hydrogen bonds with adjacent amino acids and domains. Therefore, mutations in amino acids discovered by alanine mutagenesis screening in this study may directly affect the transport of PEPT1 substrates, including cefaclor, causing significant variation in pharmacokinetic results. As a result, in relation to the interpretation of the pharmacokinetic variability of cefaclor, it will be necessary to pay attention to the polymorphism of the amino acids newly discovered in this study (identified based on alanine mutagenesis and the search for key residues in the interaction between cefaclor and PEPT1) rather than the *SLC15A1* exons 5 and 16 targeted at the beginning of this study.

### 2.5. Analysis of Genetic Polymorphism Effects Based on Molecular Dynamics Simulation

**Figure 6:**
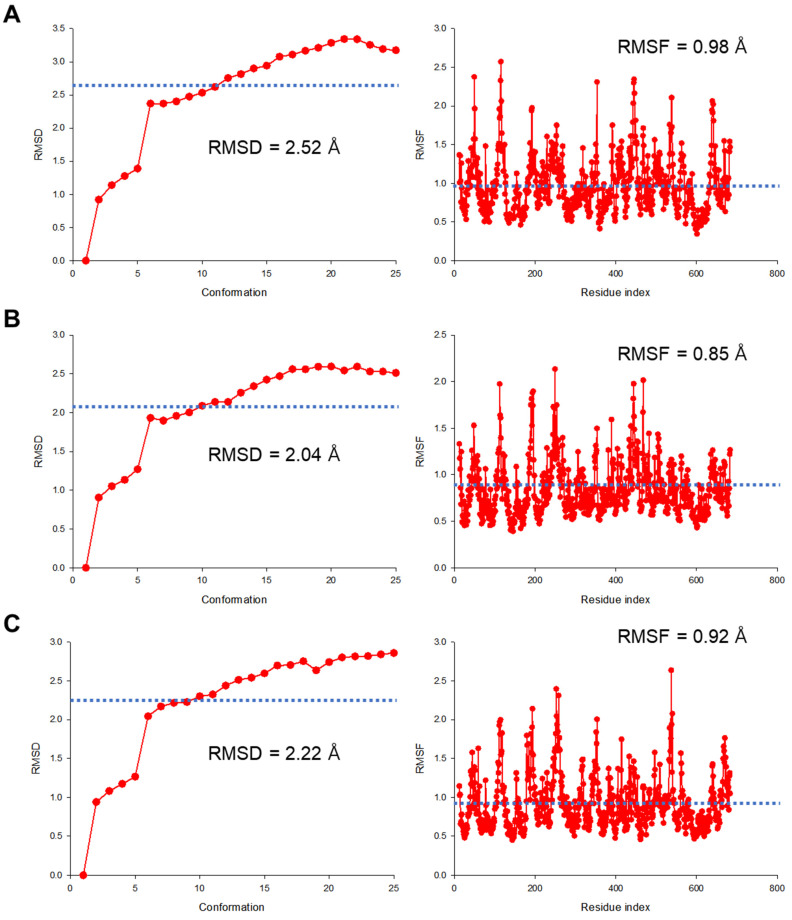

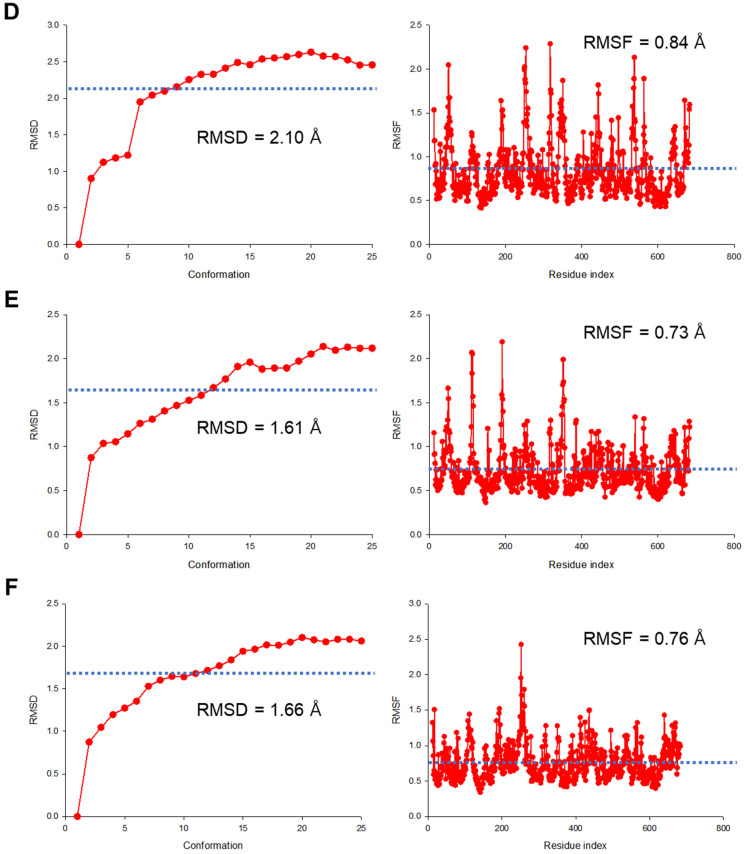
Results of structural root mean square deviation (RMSD) and root mean square fluctuation (RMSF) from molecular dynamic simulations in PEPT1 outward-facing apo-state (**A**,**D**), open conformation (**B**,**E**), and occluded conformations (**C**,**F**) according to genetic polymorphisms of *SLC15A1* exon 5 (c.381G>A; (**A**–**C**)) and 16 (c.1287G>C; (**D**–**F**)). The left and right graphs in (**A**–**F**) show the RMSD for each molecular conformation and the RMSF for each residue in the structure generated during the molecular dynamic simulations production stage, respectively. The blue horizontal dotted lines and values presented in the RMSD and RMSF graphs in (**A**–**F**) represent the average values across all molecular conformations generated during the molecular dynamic simulation production stage.

Since protein molecules are not static structures in vivo and many biological processes are mediated by molecular motion and dynamics, molecular dynamics simulations were performed to determine the movement of protein molecules in structural space over time. This was also to comprehensively determine the structural stability aspects of PEPT1 according to the genetic polymorphisms of *SLC15A1* exons 5 and 16 in terms of conformation change, domain flexibility, protein folding, and thermodynamic energy. Figure S12 shows thermodynamic structural stability energy profiles according to molecular dynamics simulations in three states of PEPT1 outward-facing, reflecting the genetic polymorphisms of *SLC15A1* exons 5 and 16. As a result, the energies (comprehensively considering interactions between molecules and the degree of structural stabilization) estimated at the production stage for all three states of PEPT1 outward-facing were lower than −176,400 kcal/mol and showed energy fluctuations within 10% around the average. In other words, in the production phase of the molecular dynamic simulation, the energy value was very stable, and no large energy fluctuations were identified around the mean value. This implied that the molecular dynamic results reconfirmed that the genetic polymorphisms of *SLC15A1* exons 5 and 16 did not have a significant structural impact on PEPT1 outward-facing states. In addition, the reason why the energy values of PEPT1 conformations derived from molecular dynamic simulations were largely lowered may be related to the structural stabilization of PEPT1 apo- and cefaclor-docked states due to interactions with the surrounding solvent and adjacent molecules. Figure 6 shows the structural dynamics profiles according to molecular dynamics simulations in three states of PEPT1 outward-facing, reflecting the genetic polymorphisms of *SLC15A1* exons 5 and 16, respectively. In all three states of PEPT1 outward-facing, the RMSD and root mean square fluctuation (RMSF) values of the protein calculated at the production stage were not high, within 3.5 and 3 Å, respectively. This suggested that the genetic polymorphisms of *SLC15A1* exons 5 and 16 did not have a significant effect on structural changes and mobilities in PEPT1 outward-facing states.

## 3. Materials and Methods

### 3.1. Research Approach

This study was largely conducted in two steps, with several detailed processes included within each step. In the first step, the in vivo pharmacokinetic diversity analysis of cefaclor was performed based on the genotyping results of *SLC15A1* exons 5 and 16. This step included analyzing pharmacokinetic profiles, calculating parameter values, comparing significance between groups, and attempting to apply covariates using a population pharmacokinetic model. In the second step, molecular modeling-based structural correlation analysis of the interaction between cefaclor and PEPT1 according to the genetic polymorphisms of *SLC15A1* exons 5 and 16 was performed. This step was performed to rationally explain at the molecular structure level (as an extension to systemic pharmacometrics research) the macroscopic pharmacokinetics results of cefaclor according to the *SLC15A1* exon 5 and 16 genetic polymorphisms identified in the previous first step. In detail, four processes were included in the second step, the first of which was to divide the conformational state of PEPT1 into three and perform structural analysis, mechanical function exploration, and interaction analysis with cefaclor in each state. The second process was to analyze changes in PEPT1 structure and interaction with cefaclor following a point mutation of *SLC15A1* exons 5 and 16. The third process was to confirm the degree of change in PEPT1 structural stability and substrate interaction with cefaclor through the application of previously reported SNPs of PEPT1 [18] and alanine mutagenesis. In addition, the fourth process was to confirm the thermodynamic structural stability and steric changes of PEPT1 according to the genetic polymorphisms of *SLC15A1* exons 5 and 16 through molecular dynamic simulations.

### 3.2. Pharmacokinetic Study

Cefaclor bioequivalence results in 24 healthy Korean men obtained from a previous study [7] were reused for the analysis of cefaclor pharmacokinetics according to PEPT1 genetic polymorphisms in this study. Among the previous bioequivalence results, those for the test formulation were excluded, and only the clinical results for the reference drug, cefaclor 250 mg capsule (Eli Lilly and Company, Indianapolis, IN, USA; Lot number: L1692Y1) were used. No notable side effects were noted in any subjects during the clinical trial. Table S2 presents the demographic information of the subjects, including physiological and biochemical parameters. The determination of clinical biochemical parameter values was performed by serological analysis through a dry automated analyzer. The analytical instrument was a microside VITROS (Ortho Clinical Diagnostics, Raritan, NJ, USA) operated by reflectance spectrophotometry. Information on the selection and progress of clinical trial participants is presented in Supplementary Information S1, and information on clinical trial design and sampling is presented in Supplementary Information S2. The clinical trial protocol was thoroughly reviewed and officially approved (approval number: 112; 09.03.2004) by the Institutional Review Board of the Institute of Bioequivalence and Bridging Study (Gwangju, Republic of Korea). Information on the high-performance liquid chromatography-ultraviolet (HPLC-UV) method used to quantify cefaclor in plasma samples is briefly presented in Supplementary Information S3.

Genetic polymorphism information for all subjects participating in the clinical trial could be obtained using blank plasma samples derived from each subject. The genetic information targeted in this study was *SLC15A1* exons 5 and 16 as the PEPT1 coding gene. SNPs in *SLC15A1* exons 5 and 16 were analyzed through pyrosequencing and restriction fragment length polymorphism (RFLP) via polymerase chain reaction (PCR), respectively. The target SNPs in *SLC15A1* exons 5 and 16 were c.381G>A and c.1287G>C, respectively, and the genetic polymorphism analysis methods for these were presented in Supplementary Information S4.

### 3.3. Pharmacokinetic Analysis

The results of the analysis of plasma concentration over time after single oral administration of cefaclor 250 mg capsule could be calculated as cefaclor pharmacokinetic parameter values through non-compartmental analysis (NCA). NCA was performed using Phoenix WinNonlin software (version 8.4, Certara Inc., Princeton, NJ, USA). The main calculated pharmacokinetic parameters are area under the curve in a plasma concentration-time graph (AUC), clearance (CL/F), peak plasma drug concentration after administration (C_max_), elimination half-life (T_1/2_), mean residence time (MRT), time to reach C_max_ (T_max_), and volume of the distribution (V/F). Specific calculation methods for pharmacokinetic parameters are presented in Supplementary Information S5. The calculated pharmacokinetic parameter values were classified according to the genetic polymorphisms of *SLC15A1* exons 5 and 16 and compared between genotype groups. Comparison of significant differences between the three genotype groups (*SLC15A1* exon 5) was performed using one-way analysis of variance (ANOVA), and comparison of significant differences between two genotype groups (*SLC15A1* exon 16) was performed using a two-tailed *t* test. All significance judgments in statistical tests were based on a *p* value of 0.05. Additionally, differences in pharmacokinetics between groups were confirmed through a graphic comparison of cefaclor plasma concentration profiles between genotypes classified according to genetic polymorphisms of *SLC15A1* exons 5 and 16.

### 3.4. Covariate Analysis Using Population Pharmacokinetic Modeling

In the established cefaclor population pharmacokinetic model structure, an attempt was made to sequentially apply the genetic covariates to each pharmacokinetic parameter in the model by treating the genotype information of *SLC15A1* exons 5 and 16 as categorical data. This was to determine whether the genetic polymorphism information of *SLC15A1* exons 5 and 16 was appropriate as an effective covariate in explaining the inter-individual pharmacokinetic diversity of cefaclor. The established cefaclor population pharmacokinetic model to attempt to reflect covariates was a one-compartment first-order absorption (with lag-time)/elimination structure. Therefore, *SLC15A1* exons 5 (c.381GG, c.381GA, and c.381AA) and 16 (c.1287GG and c.1287GC) were sequentially applied as categorical covariates to the basic structural parameters (absorption rate constant [K_a_], total distribution volume [V_t_/F], systemic clearance [CL_s_/F], and lag-time [T_lag_]) in the model. Proportional and exponential error models were applied as residual and inter-individual error models, respectively. Covariate analysis using population pharmacokinetic modeling was performed using a non-linear mixed effects model approach using Phoenix NLME (version 8.4, Certara Inc.) software. The criteria for selecting an appropriate model at each stage of covariate application were based on various statistical significance tools derived by Phoenix NLME. Akaike’s information criterion (AIC), −2LL, and GOF plots were included, and the significance of the total number of parameters applied to the model (increasing or decreasing degrees of freedom) was also considered. Significance was judged based on a *Chi*-square distribution *p* value of 0.05 (for forward selection) and 0.01 (for backward elimination) at −2LL and OFV. GOF plots included comparison of population and individual predicted concentrations by observed plasma concentration, comparison of conditional weighted residuals (CWRES) by cefaclor exposure time and population predicted concentration, and quantile-quantile analysis of CWRES.

### 3.5. Molecular Modeling-Based Cefaclor-PEPT1 Interaction Analysis

Binding of a small molecular ligand (as cefaclor) to a protein receptor molecule (as PEPT1) was performed using docking protocols in BIOVIA Discovery Studio^®^ (Dassault Systèmes, Vélizy-Villacoublay, France). The docking protocols consisted of three major steps: protein and ligand structure formation, DBP establishment, and interaction energy analysis following ligand docking. Protein and ligand structure formation was performed through clean protein, prepare protein, and prepare ligand processes, which included generation of complete residues and covalent bonds based on sequence information, generation of isomer and tautomer three-dimensional structures with optimal energy, and correction of bad valences. The outward-facing structure of PEPT1 was divided into apo-state and open and occluded conformations in detail, and all structures were generated through chemistry at Harvard Macromolecular mechanics (CHARMm)-based forcefield, and the sequence and basic structure information for these were derived from the protein data bank (PDB). The reason outward-facing apo-state (PDB ID: 7PN1), open (PDB ID: 7PMX), and occluded (PDB ID: 7PMW) conformations were targeted as PEPT1 structures in this study was because they were accessible through electron microscopy-based experimental data information as PEPT1 structures for humans [19]. The search for DBP in PEPT1 was performed by overlapping the results of the receptor cavity search based on the target ligand size and the binding domain information of the di-peptide substrate (Ala-Phe) derived from PDB [19]. Ligand binding within the searched DBP was performed via CDOCKER, a grid-based molecular docking method. The binding and interaction stability between the protein and the ligand were confirmed quantitatively through the calculation of −CDOCKER interaction energy and, at the same time, qualitatively through the analysis of non-bond interactions.

### 3.6. Structural Analysis According to Genetic Polymorphisms of SLC15A1 Exons 5 and 16

A point mutation protocol was applied to identify changes in PEPT1 function (as transport based on substrate interactions) and stability due to genetic polymorphisms in exons 5 and 16 of *SLC15A1* (the PEPT1 encoding gene) that have been suggested in past reports [17,18]. In this study, a point mutation protocol was attempted to molecularly explore whether the inter-individual pharmacokinetic variability of cefaclor, known as a PEPT1 substrate, could be explained by genetic polymorphisms in *SLC15A1* exons 5 and 16. The point mutation protocol was conducted by generating PEPT1 amino acid substitution structures according to base substitutions in exons 5 and 16 in *SLC15A1* and calculating protein energy changes according to the mutated structure. The main energies calculated through the application of the point mutation protocol were structural stability and substrate interaction energy change values. In addition, the protein structure output through the application of the point mutation protocol overlapped with the original control structure, confirming the overall degree of steric change, and through this, the structural impact of the corresponding mutation site in the protein was analyzed.

### 3.7. Structural Analysis following Application of Other Additional SLC15A1 SNPs

*SLC15A1* SNP information from a past report [18] was sequentially applied to explore the effects of changes in PEPT1 structural stability and interaction with cefaclor due to genetic polymorphisms at additional positions other than the *SLC15A1* exon 5 and 16 regions, which was the main target in this study. This was expected to increase the structural and functional understanding of PEPT1 and provide a scientific basis for genetic focus points in future studies of the pharmacokinetic diversity of substrate drugs. The applicable *SLC15A1* SNP information corresponded to missense results that cause amino acid changes in PEPT1 due to base substitution. Mutation energies were calculated by applying *SLC15A1* SNPs to all three states of PEPT1 outward-facing. Specifically, protein stability in the apo-state, changes in binding energy with cefaclor, and protein stability in the outward-facing open and occluded conformations were estimated. In addition, an amino acid substitution simulation targeting key cefaclor interaction residues in PEPT1 DBP explored in this study was additionally performed, making it possible to analyze changes in protein mutation energy.

### 3.8. Structural Analysis through Molecular Dynamics Simulation

Molecular dynamics simulations according to the genetic polymorphisms of *SLC15A1* exons 5 and 16 for all three states of PEPT1 outward-facing were conducted sequentially in six stages. In the first stage, solvation of the protein was performed, which was made possible by applying the orthorhombic cell shape of the solvent molecules as an explicit periodic boundary model (a minimum distance from the boundary of 7 Å). Since protein solvation plays an important role in the biological process, it was necessary to explain the solvent effect through the arrangement of water and ion molecules similar to the biological environment around the protein structure. In the second and third stages, the prepared structures were subjected to structural energy optimization through initial and additional minimization. The algorithms applied to the initial and additional minimization processes were steepest descent and Newton-Raphson, respectively, which are smart minimizers and correspond to a stepwise energy optimization approach. As a fourth stage, heating was performed on the energy-optimized structure and the initial and target temperatures were set to 50 and 300 K, respectively. In the fifth stage, equilibration was performed to confirm the stability of the structure; the target temperature was maintained at 300 K, and the adjusted velocity frequency was set to 50. As a sixth stage, production was performed to check the movements of the equilibrated structure over time, and the simulation and result output interval times were set to 50 and 2 ps, respectively. The ensemble type applied in the production stage was isobaric-isothermal, which was a system in which the volume and energy were changed while the number of particles, pressure, and temperature were kept constant. Additionally, the applied temperature in the production stage was controlled by a Langevin dynamics thermostat and varied within ±3 K based on the set temperature of 300 K. The molecular dynamics simulation results were interpreted through trajectory analysis, and the main focus was the comparison of the conformational frame at the production stage through RMSD and RMSF calculations. Here, RMSD and RMSF are quantitative measures of the degree of similarity and movement between generated protein structures over simulation time, respectively.

## 4. Conclusions

Analysis of cefaclor clinical pharmacokinetics according to genetic polymorphisms of *SLC15A1* exons 5 and 16 showed no significant differences between groups. Even when attempting to reflect them as candidate covariates in the cefaclor population pharmacokinetics model, the genetic polymorphisms of *SLC15A1* exons 5 and 16 were not effective in explaining the inter-individual cefaclor pharmacokinetics variability. Molecular modeling results showed that amino acid substitutions of p.Ser117Asn and p.Gly419Ala according to genetic polymorphisms in *SLC15A1* exons 5 and 16 did not significantly affect the structural stability of PEPT1 and the change in interaction with cefaclor. Also in the molecular dynamics simulation results, movements that lead to structural instability and disruption of substrate interactions were not identified. As a result, it was suggested that the interpretation of the association of genetic polymorphisms in *SLC15A1* exons 5 and 16 would not be important in explaining the variation in cefaclor pharmacokinetics within the population. Based on the results of this study, focusing on genetic polymorphisms in *SLC15A1* exons 5 and 16 is unlikely to be effective in precision medicine involving consideration of inter-individual pharmacokinetic variation in cefaclor as well as other PEPT1 substrate drugs.

## Figures and Tables

**Table 1:** Comparison of pharmacokinetic parameter values (mean ± standard deviation) of cefaclor (after oral administration of 250 mg) between groups according to *SLC15A1* exon 5 genetic polymorphisms (c.381G>A).

Parameters	Units	*SLC15A1* Exon 5		
		c.381GG (*n* = 7)	c.381GA (*n* = 12)	c.381AA (*n* = 5)
AUC_all_	h·μg/mL	7.59 ± 1.52	7.97 ± 1.83	7.81 ± 2.22
AUC_inf_	h·μg/mL	7.74 ± 1.53	8.21 ± 1.86	7.97 ± 2.20
CL/F	mL/hr	33,503.74 ± 7249.84	32,189.36 ± 8599.64	33,277.68 ± 8681.99
C_max_	μg/mL	6.51 ± 1.90	7.20 ± 2.32	5.88 ± 2.66
T_1/2_	h	0.60 ± 0.06	0.70 ± 0.20	0.62 ± 0.16
MRT	h	1.26 ± 0.19	1.43 ± 0.40	1.64 ± 0.34
T_max_	h	0.82 ± 0.35	0.77 ± 0.31	0.95 ± 0.11
V/F	mL	29,008.13 ± 5949.24	31,689.12 ± 9513.51	29,598.65 ± 10,516.69

AUC_all_, area under the curve (in a plasma concentration-time graph) from 0 h to the last measured time; AUC_inf_, area under the curve (in a plasma concentration-time graph) from 0 h to infinite; CL/F, clearance; C_max_, peak plasma drug concentration after administration; T_1/2_, elimination half-life; MRT, mean residence time; T_max_, time to reach C_max_; V/F, volume of the distribution; Parameter values were calculated through non-compartmental analysis.

**Table 2:** Comparison of pharmacokinetic parameter values (mean ± standard deviation) of cefaclor (after oral administration of 250 mg) between groups according to *SLC15A1* exon 16 genetic polymorphisms (c.1287G>C).

Parameters	Units	*SLC15A1* Exon 16	
		c.1287GG (*n* = 22)	c.1287GC (*n* = 2)
AUC_all_	h·μg/mL	7.92 ± 1.77	6.85 ± 1.69
AUC_inf_	h·μg/mL	8.11 ± 1.79	7.02 ± 1.69
CL/F	mL/hr	32,444.65 ± 7951.04	36,702.30 ± 8866.25
C_max_	μg/mL	6.77 ± 2.34	6.20 ± 0.23
T_1/2_	h	0.67 ± 0.17	0.53 ± 0.11
MRT	h	1.44 ± 0.37	1.26 ± 0.09
T_max_	h	0.82 ± 0.30	0.88 ± 0.18
V/F	mL	30,769.65 ± 8896.89	27,193.69 ± 1112.81

AUC_all_, area under the curve (in a plasma concentration-time graph) from 0 h to the last measured time; AUC_inf_, area under the curve (in a plasma concentration-time graph) from 0 h to infinite; CL/F, clearance; C_max_, peak plasma drug concentration after administration; T_1/2_, elimination half-life; MRT, mean residence time; T_max_, time to reach C_max_; V/F, volume of the distribution; Parameter values were calculated through non-compartmental analysis.

**Table 3:** Structural stability and binding (with cefaclor) energy analysis results according to application (step-by-step mutation progression at each point) of previously reported *SCL15A1* single nucleotide polymorphisms (SNP) in three conformations (apo-state, open conformation, and occluded conformation) of PEPT1 outward-facing.

Exon	SNP No.	Nucleotide Change ^a^	Amino Acid Change	Outward-Facing Apo-State (Stability Energy [kcal/mol], pH 7.4)	Outward-Facing Open Conformation (Stability Energy [kcal/mol], pH 7.4)	Outward-Facing Occluded Conformation (Stability Energy [kcal/mol], pH 7.4)	Outward-Facing Open Conformation (Binding Energy [kcal/mol], pH 7.4)	Outward-Facing Occluded Conformation (Binding Energy [kcal/mol], pH 7.4)
3	2	G>A	p.Val21Ile	−0.94 (stabilizing) ^a^	−0.78 (stabilizing) ^b^	−0.89 (stabilizing) ^b^	0.11 (neutral) ^b^	−0.21 (neutral) ^b^
3	3	T>A	p.Phe28Tyr	0.08 (neutral) ^a^	0.63 (destabilizing) ^b^	0.25 (neutral) ^b^	0.04 (neutral) ^b^	−0.33 (neutral) ^b^
5	4	G>A	p.Ser117Asn	−0.23 (neutral) ^a^	−0.64 (stabilizing) ^b^	−0.45 (neutral) ^b^	0.00 (neutral) ^b^	0.01 (neutral) ^b^
5	5	C>A	p.Ser117Arg	−1.74 (stabilizing) ^a^	−2.07 (stabilizing) ^b^	−1.03 (stabilizing) ^b^	−0.01 (neutral) ^b^	−0.09 (neutral) ^b^
5	6	G>A	p.Val122Met	−0.62 (stabilizing) ^a^	0.18 (neutral) ^b^	0.33 (neutral) ^b^	0.11 (neutral) ^b^	−0.10 (neutral) ^b^
16	3	G>C	p.Gly419Ala	1.36 (destabilizing) ^a^	1.62 (destabilizing) ^b^	1.48 (destabilizing) ^b^	0.01 (neutral) ^b^	0.01 (neutral) ^b^
17	2	G>A	p.Val450Ile	−1.14 (stabilizing) ^a^	−0.66 (stabilizing) ^b^	−1.00 (stabilizing) ^b^	0.09 (neutral) ^b^	0.01 (neutral) ^b^
17	3	C>A	p.Thr451Asn	0.13 (neutral) ^a^	0.31 (neutral) ^b^	−0.05 (neutral) ^b^	−0.01 (neutral) ^b^	0.00 (neutral) ^b^
20	3	C>T	p.Pro537Ser	−0.04 (neutral) ^a^	0.90 (destabilizing) ^b^	0.61 (destabilizing) ^b^	0.01 (neutral) ^b^	−0.10 (neutral) ^b^

^a^ Among the previously reported SNPs in the *SLC15A1* gene [18], these refer to those that cause amino acid substitutions. ^b^ The mutation effects were determined based on mutation energy values of ±0.50 kcal/mol.

**Table 4:** Structural binding (with cefaclor) energy analysis results according to application (step-by-step mutation progression at each point) of point mutations of key interaction residues between PEPT1 and cefaclor explored in this study in open and occluded conformations of PEPT1 outward-facing.

Key Residues Interacting with Cefaclor in Outward-Facing Open Conformation ^a^	Amino Acid Change ^b^	Outward-Facing Open Conformation (Binding Energy [kcal/mol], pH 7.4)	Key Residues Interacting with Cefaclor in Outward-Facing Occluded Conformation ^a^	Amino Acid Change ^b^	Outward-Facing Occluded Conformation (Binding Energy [kcal/mol], pH 7.4)
27 Arg	p.Arg27Glu	2.22 (destabilizing) ^c^	27 Arg	p.Arg27Thr	1.15 (destabilizing) ^c^
31 Tyr	p.Tyr31Gly	1.95 (destabilizing) ^c^	31 Tyr	p.Tyr31Gly	1.46 (destabilizing) ^c^
171 Asn	p.Asn171Phe	2.48 (destabilizing) ^c^	171 Asn	p.Asn171Trp	5.35 (destabilizing) ^c^
297 Phe	p.Phe297Gly	0.75 (destabilizing) ^c^	297 Phe	p.Phe297Gly	0.90 (destabilizing) ^c^
329 Asn	p.Asn329Gly	0.69 (destabilizing) ^c^	329 Asn	p.Asn329Ile	1.80 (destabilizing) ^c^
140 Lys	p.Lys140Glu	1.50 (destabilizing) ^c^	64 Tyr	p.Tyr64Asp	0.46 (neutral) ^c^
330 Ala	p.Ala330Tyr	1.73 (destabilizing) ^c^	167 Tyr	p.Tyr167Thr	0.72 (destabilizing) ^c^

^a^ In each conformation of PEPT1 outward-facing, the interaction key residues were defined according to the cefaclor docking simulation within the drug binding pocket (DBP) performed in this study. ^b^ This means that only the results confirming the highest energy increase according to sequential substitutions of 20 amino acids are presented. ^c^ The mutation effects were determined based on mutation energy values of ±0.50 kcal/mol.

**Table 5:** Position and energy information for the top five amino acids causing structural instability derived after performing alanine mutagenesis in PEPT1 outward-facing apo-state, open conformation, and occluded conformations.

Amino Acid Change Site ^a^	Outward-Facing Apo-State (Stability Energy [kcal/mol], pH 7.4)	Amino Acid Change Site ^a^	Outward-Facing Open Conformation (Stability Energy [kcal/mol], pH 7.4)	Amino Acid Change Site ^a^	Outward-Facing Occluded Conformation (Stability Energy [kcal/mol], pH 7.4)
622 Trp	4.90 (destabilizing) ^b^	264 Trp	5.25 (destabilizing) ^b^	223 Tyr	5.24 (destabilizing) ^b^
223 Tyr	4.91 (destabilizing) ^b^	622 Trp	5.31 (destabilizing) ^b^	622 Trp	5.41 (destabilizing) ^b^
264 Trp	5.29 (destabilizing) ^b^	294 Trp	5.53 (destabilizing) ^b^	304 Trp	6.02 (destabilizing) ^b^
465 Trp	5.47 (destabilizing) ^b^	135 Gly	6.21 (destabilizing) ^b^	313 Gly	6.36 (destabilizing) ^b^
304 Trp	7.02 (destabilizing) ^b^	304 Trp	6.38 (destabilizing) ^b^	135 Gly	7.21 (destabilizing) ^b^

^a^ This refers to the positions of the top five amino acids causing structural instability derived after performing alanine mutagenesis in each state of PEPT1 outward-facing. ^b^ The mutation effects were determined based on mutation energy values of ±0.50 kcal/mol.

## Data Availability

Data relevant to this study are accessible in the manuscript and Supplementary Materials.

## Supplementary Materials

The following supporting information can be downloaded at: https://www.mdpi.com/article/10.3390/ijms25136880/s1.
