# Supplementary material for: Structure-Based Analysis of Cefaclor Pharmacokinetic Diversity According to Human Peptide Transporter-1 Genetic Polymorphism
Source: Int J Mol Sci. 2024 Jun 22;25(13):6880. doi: 10.3390/ijms25136880 (PMC11241437; doi:10.3390/ijms25136880)
Supplement: Supplementary file 1 [file ijms-25-06880-s001.zip › ijms-3046126-supplementary.pdf]

Structure-based analysis of cefaclor pharmacokinetic diversity according to human peptide transporter-1 genetic polymorphism

Supplementary Information & Tables & Figures (with captions)

Ji-Hun Jang [a] and Seung-Hyun Jeong [a, b, *]

[a] *College of Pharmacy, Sunchon National University, 255 Jungang-ro, Suncheon-Si, Jeollanam-do, 57922, Republic of Korea*

[b] *College of Pharmacy and Research Institute of Life and Pharmaceutical Sciences, Sunchon National University, Suncheon-Si 57922, Republic of Korea*

* Corresponding author:

Prof. Seung-Hyun Jeong

Department of Pharmacy, College of Pharmacy, Sunchon National University, 255 Jungang-ro, Suncheon-Si, Jeollanam-do, 57922, Republic of Korea

E-mail address: jeongsh@scnu.ac.kr (S.-H. Jeong)

Supplementary Information S1

Subjects

The number of subjects recruited to conduct this clinical trial was 32, of which 24 who met the selection criteria and did not meet the exclusion criteria participated in the final trial. Participant selection criteria were as follows: Age at screening 19 years or older; Body mass index (BMI) is in the range of 18-30 kg/m^2; Weight more than 50 kg; No clinically significant congenital or chronic diseases and no pathological symptoms or findings as a result of medical examination; Diagnostic tests (blood and urine tests) and electrocardiogram results were within normal values. The exclusion criteria were as follows: Have taken drugs that induce or inhibit drug-metabolizing enzymes, such as barbiturates, within 30 days before the test date, or have taken drugs that may affect the test within 10 days; Have a history of gastrointestinal resection, which may affect drug absorption; Excessive drinking within 1 month prior to test date; Hypersensitivity to the test drug or its components (especially cephalosporins and beta-lactams); Clinical history of mental illness. All 24 people who participated in the trial underwent thorough health examinations (vital signs, physical examination, electrocardiogram, hematology test, blood chemistry test, urine test, serology test, etc.), no clinically significant adverse reactions such as abnormal values or findings were identified, and no concomitant medications were administered. All subjects provided written informed consent before participating in the clinical trial. This clinical study was conducted in accordance with the Declaration of Helsinki as implemented in the Good Clinical Practice guidelines. And the clinical trial was conducted at the Chonnam National University Clinical Trial Center (Gwangju, Republic of Korea) and the total duration was from 3 September, 2004 to 12 September, 2004. This included the follow-up period of subjects from the start of cefaclor administration to the end of treatment.

Supplementary Information S2

Clinical trial design and sampling

This bioequivalence study was performed as randomized, single-dose, open-label, crossover, and two-way studies (with a washout period of 7 days). Prior to the clinical trial, subjects had a heparin-locked (150 unit/mL) JELCO 22G angio-catheter (Smiths Medical, Minneapolis, MN, USA) installed in a vein on the arm or back of the hand, and 6 mL of blank blood was collected. All subjects were then given a single dose of cefaclor capsule 250 mg orally with 150 mL of water. Subjects were fasted for more than 10 h before administration. Blood sampling from subjects was performed at a total of 11 points post-dose and the specific times were as follows: Before administration (0 h) and at 0.25, 0.5, 0.75, 1, 1.5, 2, 2.5, 3, 4, 5, and 6 h after oral administration. As for the blood collection method, in order to completely remove the heparinized saline solution remaining in the venous catheter during blood collection, approximately 1 mL of blood was collected and discarded each time, and approximately 6 mL of blood was then collected. The collected blood was placed in sodium heparinized vacuum tubes (Becton Dickinson, Franklin Lakes, NJ, USA) with the subject control number and blood collection time written on them. After each blood draw, saline solution containing heparin for injection was administered to prevent clotting of the blood remaining in the intravenous catheter. The collected blood was centrifuged at 3,000 × *g* for 10 min in a centrifuge set at 4°C, and then about 1 mL of plasma was taken and transferred to an Eppendorf tube (Eppendorf, Hamburg, Germany) and stored at −80°C until analysis.

Supplementary Information S3

Determination of cefaclor plasma concentrations

Concentrations of unchanged cefaclor in plasma were determined by applying high performance liquid chromatography coupled with an ultraviolet (HPLC-UV), which has been established and validated in previous studies. The LC10 ADvp system (Shimadzu Inc., Kyoto, Japan) was used as an analysis device. Briefly describing the analysis method, plasma samples were pretreated through liquid-liquid extraction using 6% (v/v) trichloroacetic acid and then selectively separated on a Symmetry C_{18} column (5 μm, 4.6 × 150 mm; Waters Co., Milford, MA, USA) using water (mobile phase A) and methanol (mobile phase B) as mobile phases. The composition ratio of mobile phases A and B was isocratically eluted at 88:12 (v/v), and the quantification of cefaclor was performed at a wavelength of 265 nm. Cephradine, which has a similar structure to cefaclor, was used as an internal standard (IS), and the retention times in the column for cefaclor and IS were 15 min and 23 min, respectively. The lower limit of quantification was 0.1 μg/mL, and the linear calibration curve was secured with a coefficient of determination of 0.99 or higher up to the concentration range of 10 μg/mL. The concentration values of all plasma samples did not exceed 10 μg/mL, the upper limit of the calibration curve. The intra- and inter-day precision and accuracy of the method were all within 15% of the correlation coefficient, and no significant carryover was identified. The stabilities of cephradine and IS in plasma samples were all within 85-115%, showing no problems.

Supplementary Information S4

Genetic polymorphism analysis of *SLC15A1* exons 5 and 16

Single nucleotide polymorphisms (SNPs; c.381G>A) in the *SLC15A1* exon 5 were genotyped by using pyrosequencing analysis with polymerase chain reaction (PCR). Forward primer of 5′-ACCTCAGTAAGCTCCATTAATGACCTC-3′, reverse primer of 5′-CATCAAATGCGCACAAGG-3′, and sequencing primer of 5′-TGATGGCACCCCCGAC-3′ were used for *SLC15A1* exon 5 genotypes. Forward primer has a 5′ biotin-triethylene glycol label necessary for post PCR processing. PCR was performed in 20 μL reaction mixture including 1 μL extracted DNA, 1 μL of 10 pmol each primer (forward and reverse), and 17 μL autoclaved distilled water. The PCR program comprised of an initial denaturation at 95°C for 5 min followed by 35 cycles of denaturation at 95°C for 20 sec, annealing for at 50°C 30 sec, and an extension at 72°C for 20 sec. The final extension step was performed at 72°C for 5 min. The biotinylated PCR products were immobilized on streptavidin-coated Sepharose beads (Amersham Biosciences, Uppsala, Sweden). A total of 37 μL of binding buffer (10 mM Tris HCl, 2 M sodium chloride, 1 mM EDTA, 0.1% Tween 20, pH 7.6; Pyrosequencing AB, Uppsala, Sweden), 3 μL of streptavidin-coated Sepharose beads, and 20 μL of water were added to 20 μL PCR product; then, the solution was vigorously shaken for 10 min at room temperature. A 96 pin magnetic tool (Pyrosequencing AB) was used to transfer up to 96 samples at a time to solutions as follows. The beads with bound template were first transferred to 70% ethanol solution and 0.2 N sodium hydroxide solution, then to 1× washing buffer (Pyrosequencing AB), and finally into a solution of 1× annealing buffer (20 mM Tris-acetate, 2 mM magnesium acetate, pH 7.6), including the appropriate sequencing primer of 10 pmol. Lastly, this mixture

was heated for 1 min to 95 °C and then cooled to 50 °C and incubated at room temperature for at least 5 min to bind the sequencing primer to the template. After template preparation, a 96-well plate including the samples was loaded into the instrument (PSQ 96MA; Pyrosequencing AB) along with the optimal reagents (Pyro Gold; Biotage AB, Uppsala, Sweden). This instrument sequences the templates by dispensing reagents and deoxynucleotide triphosphates in a user-defined order, achieving real-time sequencing by synthesis in an automated fashion. This is achieved by creating and monitoring an enzyme cascade initiated by nucleotide incorporation that produces light emission. Pyrosequencing data were obtained by using Peak Height Determination Software (version 2.1, Pyrosequencing AB).

SNPs (c.1287G>C) in the *SLC15A1* exon 16 were genotyped by using PCR-restriction fragment length polymorphism (RFLP). Forward primer of 5′-CCCTTGTCAGGGTTAAGATGA-3′ and reverse primer of 5′-GCTTCTCTAAATCCTATTATAACAGGG-3′ were used for *SLC15A1* exon 16 genotypes. PCR was performed in 20 μL reaction mixture including 1 μL extracted DNA, 1 μL of 10 pmol each primer (forward and reverse), and 17 μL autoclaved distilled water. The PCR program comprised of an initial denaturation at 95°C for 5 min followed by 35 cycles of denaturation at 95°C for 20 sec, annealing for at 54.5°C 30 sec, and an extension at 72°C for 20 sec. The final extension step was performed at 72°C for 5 min. DNA fragments amplified by PCR were reacted at 37°C for 1 h with restriction enzyme *Sau961*, which can recognize and cleave specific sequences (only the G allele is cleaved by the *Sau961* reaction). The digested fragments were separated by electrophoresis in 2.5% agarose gel, and were visualized under ultraviolet light after staining the gel with ethidium bromide for 30 min.

Supplementary Information S5

Calculation of pharmacokinetic parameters through non-compartment analysis

The area under the curve (in a plasma concentration-time graph) from 0 h to infinite (AUC_{inf}) was calculated as the sum of area under the curve from 0 h to last measured time (AUC_{all}) and C_{last}/k, where C_{last} is the final measured concentration, t is the time in C_{last}, and k is the elimination rate constant at terminal phase. AUC_{all} was calculated using a linear trapezoidal rule from 0 to t h after oral administration of cefaclor 250 mg capsule. The area under the first-order moment curve (AUMC) was calculated as the area of the graph under the product of time and cefaclor plasma concentration over time. The mean residence time (MRT) was obtained as the ratio of AUMC and AUC_{inf}. The half-life ($T_{1/2}$) was calculated as 0.693/k, and the volume of the distribution (V/F) was calculated as dose/k · AUC_{inf}. The clearance (CL/F) was calculated by dividing the dose of cefaclor by AUC_{inf}, where F is the bioavailability of oral administration. The peak plasma drug concentration after dosing (C_{max}) and time to reach C_{max} (T_{max}) were determined from the plasma cefaclor concentration-time curves of each individual after oral administration of cefaclor 250 mg capsule.

Table S1. Model formula information according to attempts to reflect genetic polymorphisms in *SLC15A1* exons 5 and 16 as candidate covariates in the cefaclor population pharmacokinetic model.

Covariate reflection formula
K_a = tvK_a * (1 + dK_ad*SLC15A1*exon5 * [c.381G>A = 0, 1, 2][a]) * (1 + dK_ad*SLC15A1*exon16 * [c.1287G>C = 0, 1][b]) * exp(ηK_a)
V_t/F = tvV_t/F * (1 + dV_t/Fd*SLC15A1*exon5 * [c.381G>A = 0, 1, 2][a]) * (1 + dV_t/Fd*SLC15A1*exon16 * [c.1287G>C = 0, 1][b]) * exp(ηV_t/F)
CL_s/F = tvCL_s/F * (1 + dCL_s/Fd*SLC15A1*exon5 * [c.381G>A = 0, 1, 2][a]) * (1 + dCL_s/Fd*SLC15A1*exon16 * [c.1287G>C = 0, 1][b]) * exp(ηCL_s/F)
T_{lag} = tvT_{lag} * (1 + dT_{lag}d*SLC15A1*exon5 * [c.381G>A = 0, 1, 2][a]) * (1 + dT_{lag}d*SLC15A1*exon16 * [c.1287G>C = 0, 1][b]) * exp(ηT_{lag})

[a] For GG, GA, and AA, 0, 1, and 2 are reflected respectively.

[b] For GG and GC, 0 and 1 are reflected respectively.

tv, typical value; η, random variable for individual (normally distributed with mean 0 and variance ω^2); K_a, absorption rate constant; V_t/F, total distribution volume; CL_s/F, systemic clearance; T_{lag}, lag-time.

dK_ad*SLC15A1*exon5, dV_t/Fd*SLC15A1*exon5, dCL_s/Fd*SLC15A1*exon5, and dT_{lag}d*SLC15A1*exon5 refer to the degree of correlation between the genetic polymorphism of *SLC15A1* exon 5 and K_a, V_t/F, CL_s/F, and T_{lag}, respectively.

dK_ad*SLC15A1*exon16, dV_t/Fd*SLC15A1*exon16, dCL_s/Fd*SLC15A1*exon16, and dT_{lag}d*SLC15A1*exon16 refer to the degree of correlation between the genetic polymorphism of *SLC15A1* exon 16 and K_a, V_t/F, CL_s/F, and T_{lag}, respectively.

Table S2. Subjects' demographic information (n = 24).

Physiological and biochemical parameters	Units	Mean ± Standard deviation
Age	Year	22.96 ± 1.49
Weight	kg	67.02 ± 7.75
Height	cm	174.82 ± 6.48
Body surface area (BSA)[a]	m^2	1.81 ± 0.12
Body mass index (BMI)[b]	kg/m^2	22.00 ± 2.30
Albumin	g/dL	4.85 ± 0.18
Total proteins	g/dL	7.37 ± 0.30
Blood urea nitrogen	mg/dL	12.20 ± 3.60
Total bilirubin	mg/dL	1.02 ± 0.41
Cholesterol	mg/dL	159.83 ± 27.18
Alanine transaminase	U/L	17.04 ± 7.49
Aspartate transaminase	U/L	17.17 ± 4.53
Alkaline phosphatase	U/L	67.83 ± 13.85
Creatinine	mg/dL	0.97 ± 0.10
Creatinine clearance (CrCL)[c]	mL/min	112.92 ± 14.20

[a] BSA was determined on the basis of the Monsteller equation as follows: $\sqrt{height\ (cm) \times weight\ (kg)/3600}$

[b] BMI was calculated as follows: body weight (kg)/height^2 (m^2)

[c] CrCL was determined on the basis of the Cockcroft–Gault equation as follows: [(140−age) × body weight (kg)]/[serum creatinine (mg/dL) × 72].

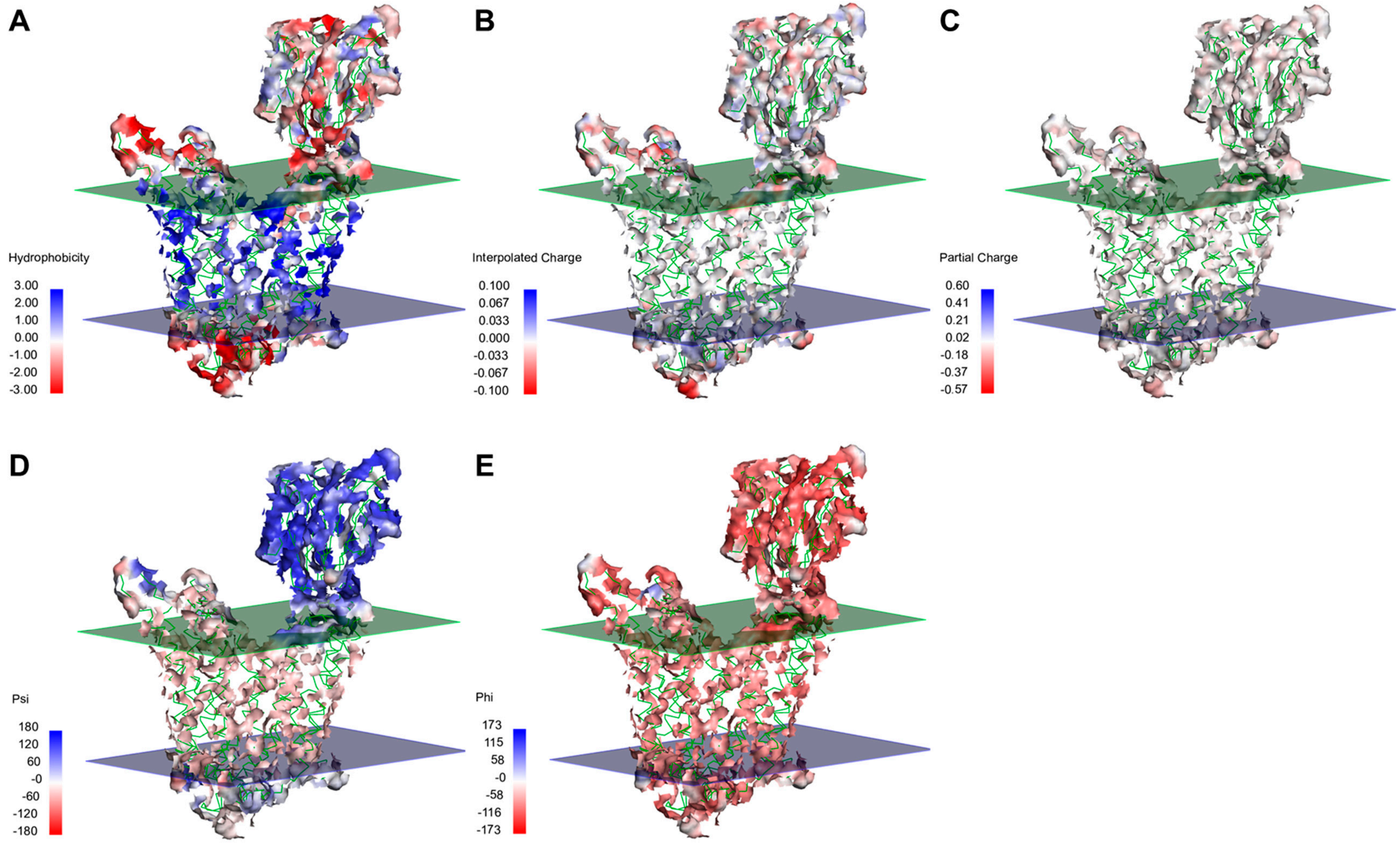

Figure S1. Analysis of structural physicochemical properties (hydrophobicity surface [A], interpolated charge surface [B], partial charge surface [C], *Psi* surface [D], and *Phi* surface [E]) of PEPT1 outward facing apo-state.

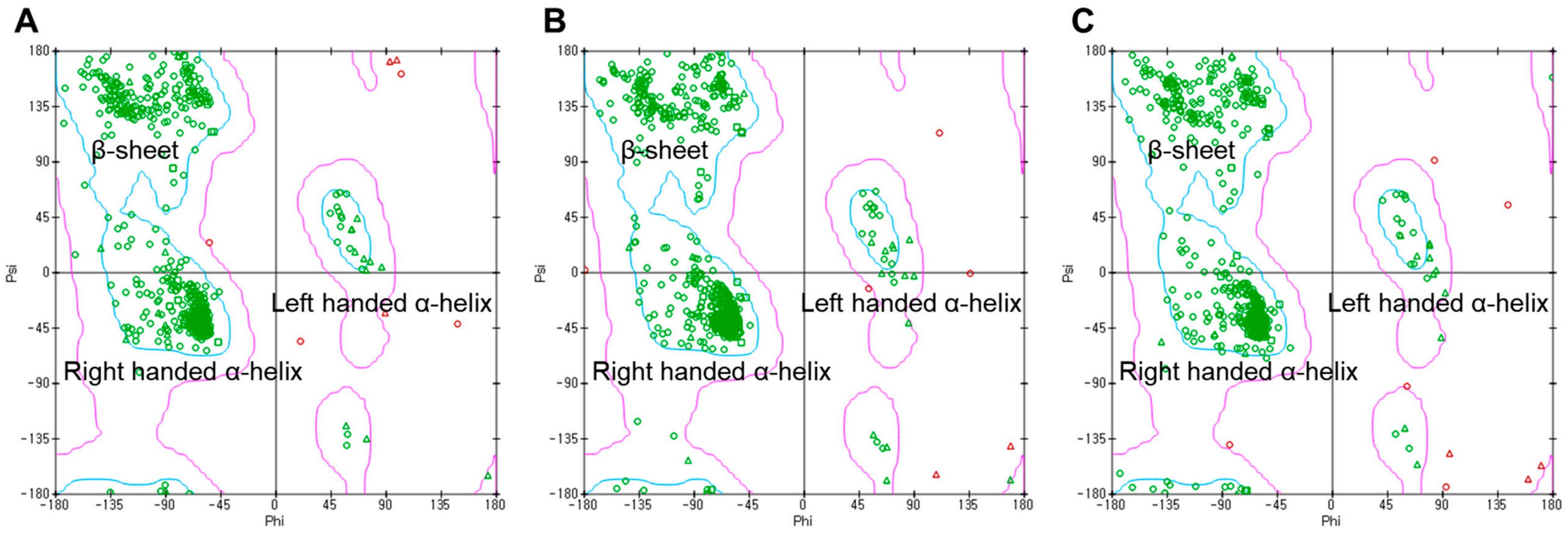

Figure S2. Ramachandran plots for the three outward facing conformations (apo-state [A], open conformation [B], and occluded conformation [C]) of PEPT1. The local backbone conformation of each PEPT1 residue is expressed graphically. Green dots in the plot indicate inclusion in the β-sheet, right handed α-helix, and left handed α-helix regions, and red dots indicate outside from those regions. The blue and purple solid lines in the plot represent fully allowed regions and additionally allowed regions, respectively. Triangular dots in the plot represent proline residues and circled dots represent other residues.

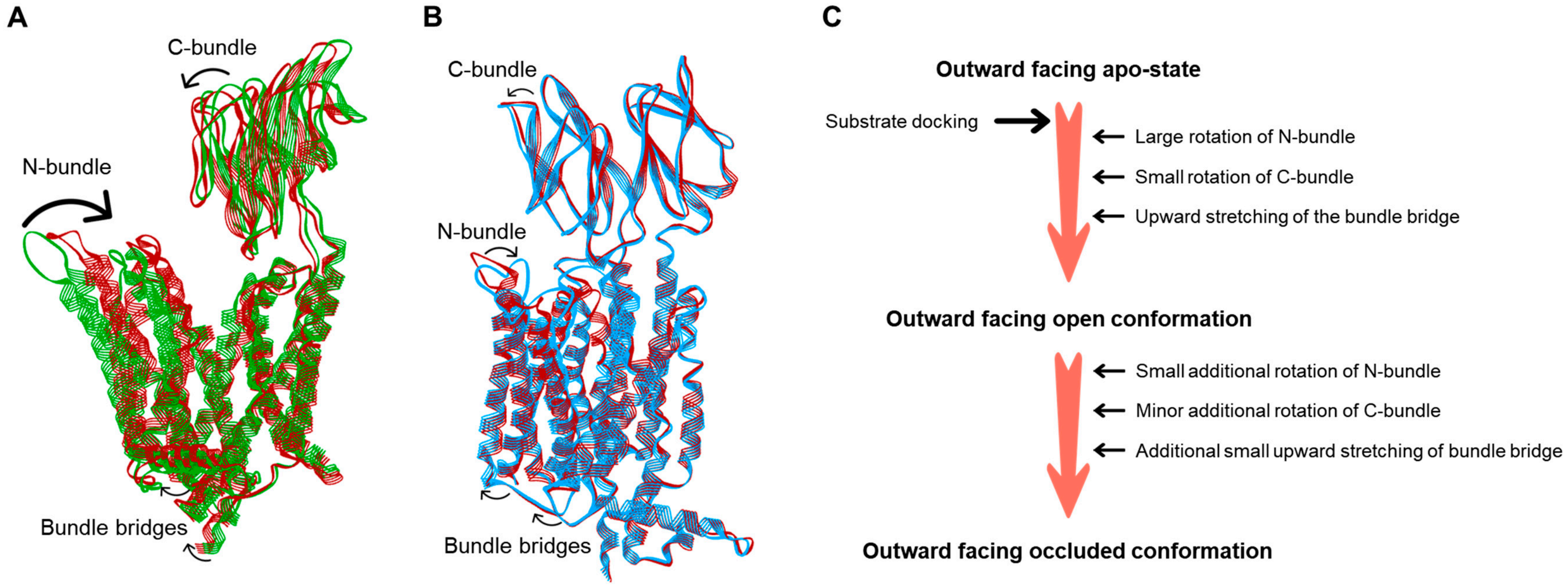

Figure S3. Analysis of the operating mechanism through structural comparison between the three outward facing conformations of PEPT1 (change from outward facing apo-state to open conformation [A], change from outward facing open conformation to occluded conformation [B], schematic diagram summarizing changes between each conformation [C]). Proteins were presented in a line ribbon display style, with green, red, and blue indicating outward facing apo-state, open conformation, and occluded conformation, respectively. The curved arrows in the structure (A and B) represent the regions where changes occur based on the previous conformation of PEPT1.

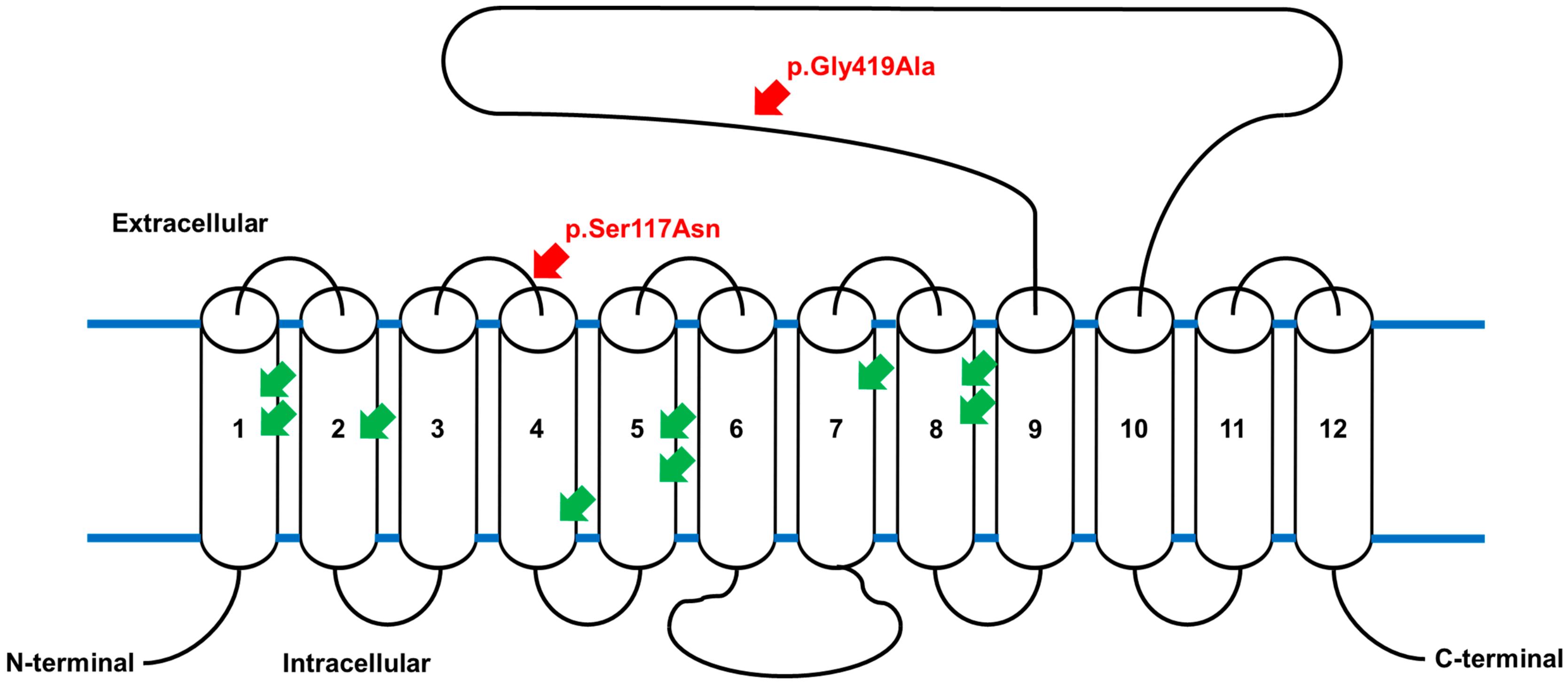

Figure S4. Predicted topology diagram of PEPT1. The cylinders in the figure represent transmembrane helices, and the black and blue solid lines represent loop connections between transmembrane helices and cell membrane boundaries, respectively. Red arrows in the figure show the locations of point mutations in *SLC15A1* exons 5 and 16. Green arrows in the figure show the positions of residues involved in the key interaction between PEPT1 and cefaclor in the open and occluded conformations of PEPT1 outward facing.

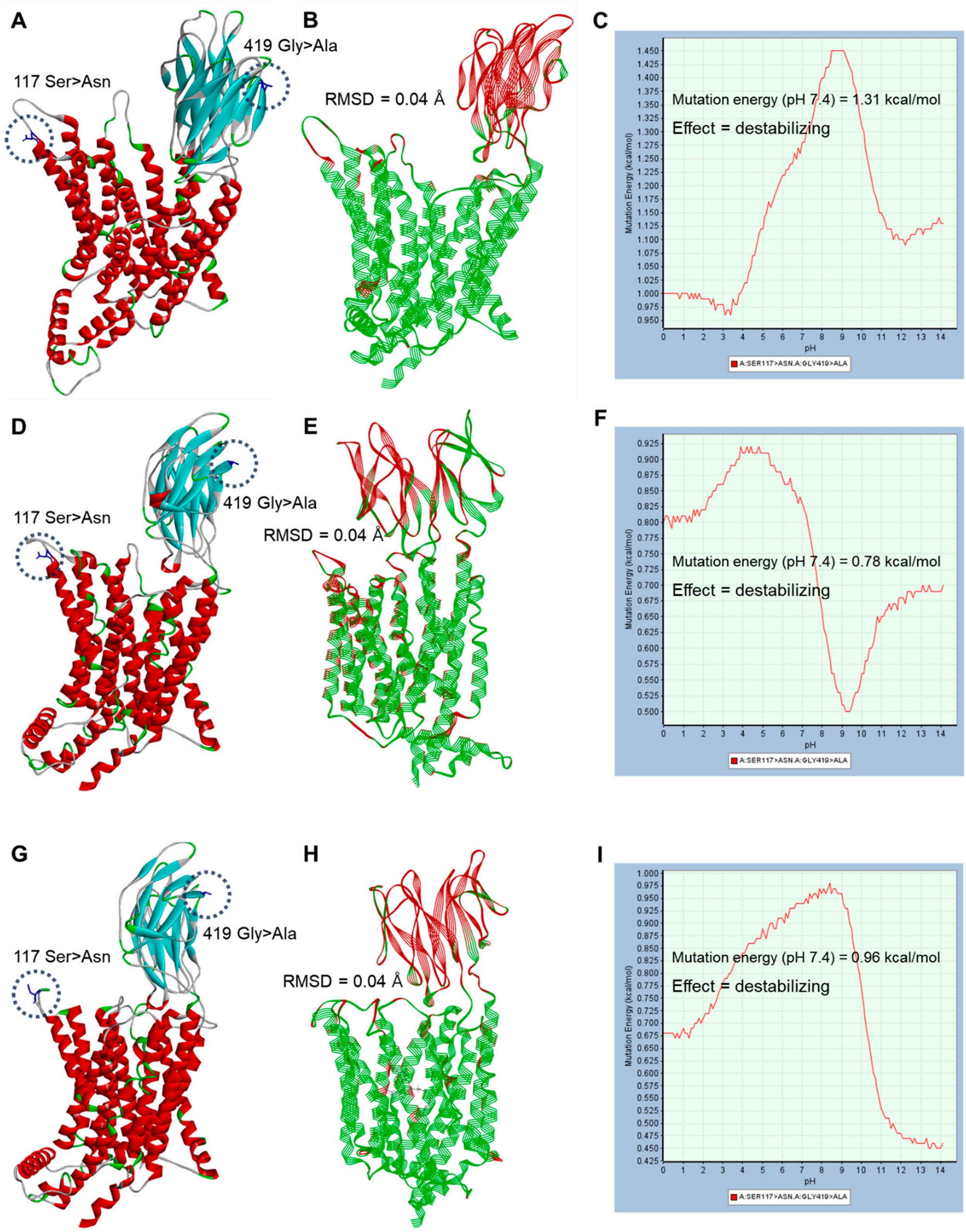
A
117 Ser>Asn
419 Gly>Ala
B
RMSD = 0.04 Å
C
Mutation energy (pH 7.4) = 1.31 kcal/mol
Effect = destabilizing
Mutation Energy (kcal/mol)
pH
A:SER117>ASN.A:GLY419>ALA
D
117 Ser>Asn
419 Gly>Ala
E
RMSD = 0.04 Å
F
Mutation energy (pH 7.4) = 0.78 kcal/mol
Effect = destabilizing
Mutation Energy (kcal/mol)
pH
A:SER117>ASN.A:GLY419>ALA
G
117 Ser>Asn
419 Gly>Ala
H
RMSD = 0.04 Å
I
Mutation energy (pH 7.4) = 0.96 kcal/mol
Effect = destabilizing
Mutation Energy (kcal/mol)
pH
A:SER117>ASN.A:GLY419>ALA

Figure S5. Structural analysis of amino acid substitution results (p.Ser117Asn and p.Gly419Ala) according to genetic polymorphisms of *SLC15A1* exon 5 and 16 (c.381G>A and c.1287G>C) in three outward facing conformations (apo-state [A-C], open conformation [D-F], and occluded conformation [G-I]) of PEPT1. Blue dotted lines in the structure indicate amino acid substitution sites in each conformation. Point mutated amino acid residues are indicated as stick type atoms. B, E, and H show graphical comparisons and root mean square deviation (RMSD) results through align and superimpose between wild-type (green colored line ribbon display style) and point mutation structures (red colored line ribbon display style). C, F, and I show the pH-dependent mutation energy (as stability) profile of PEPT1 following point mutation. The mutation effects (C, F, and I) were determined based on mutation energy values of ±0.50 kcal/mol.

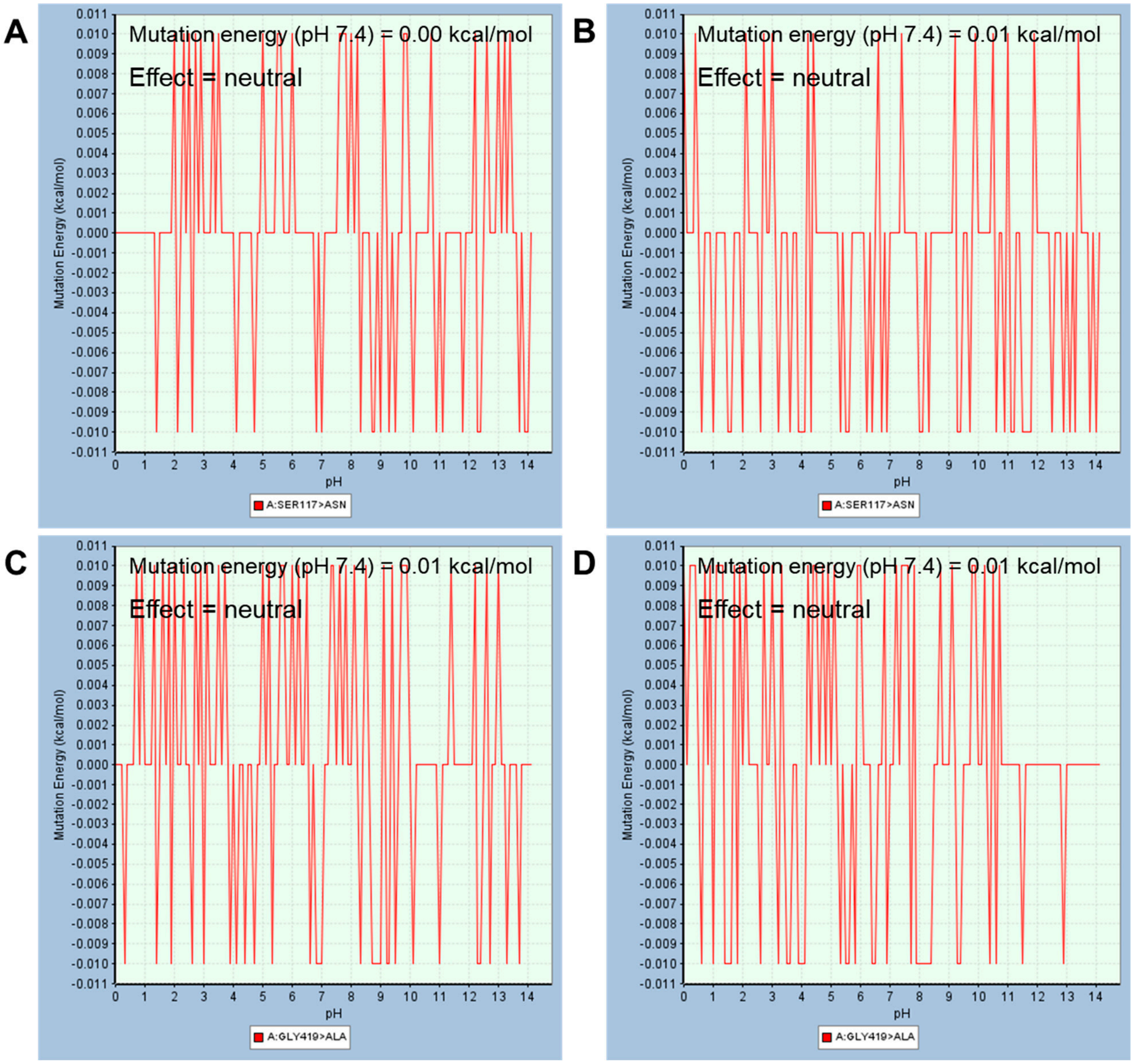

Figure S6 (continued)

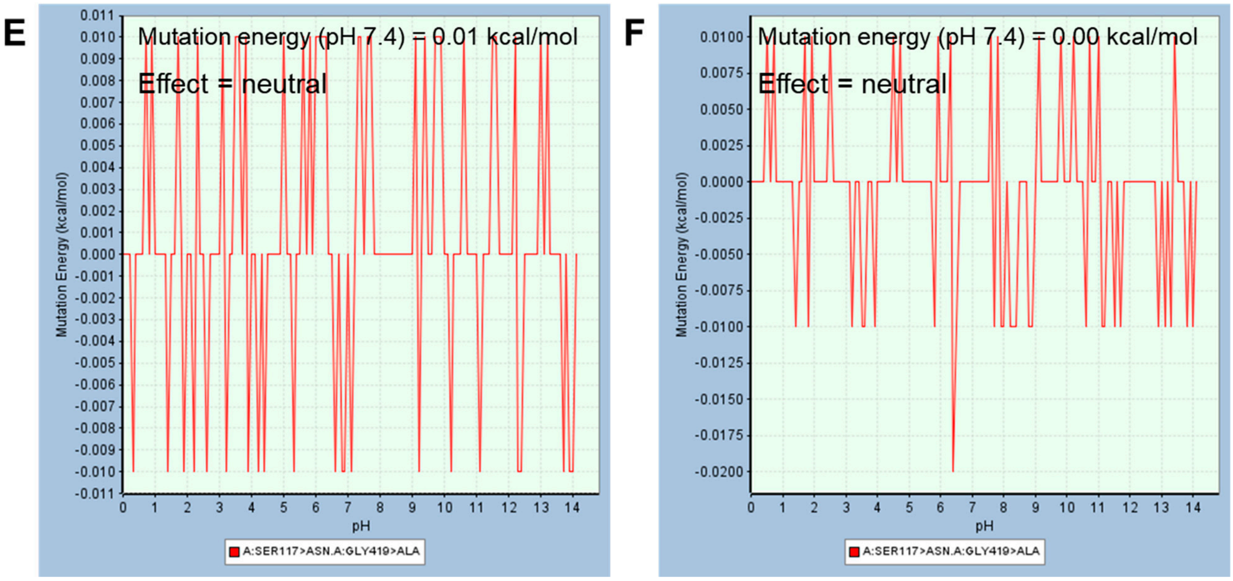

Figure S6. pH-dependent mutation energy (as an interaction with cefaclor) profiles of PEPT1 resulting from amino acid substitutions (p.Ser117Asn and/or p.Gly419Ala) in outward facing open (A, C, and E) and occluded (B, D, and F) conformations of PEPT1 according to genetic polymorphisms in exon 5 (c.381G>A; A and B) and/or 16 (c.1287G>C; C and D) of *SLC15A1*. E and F refer to the results when genetic polymorphisms occurred simultaneously in *SLC15A1* exons 5 and 16. The mutation effects (A-F) were determined based on mutation energy values of ±0.50 kcal/mol.

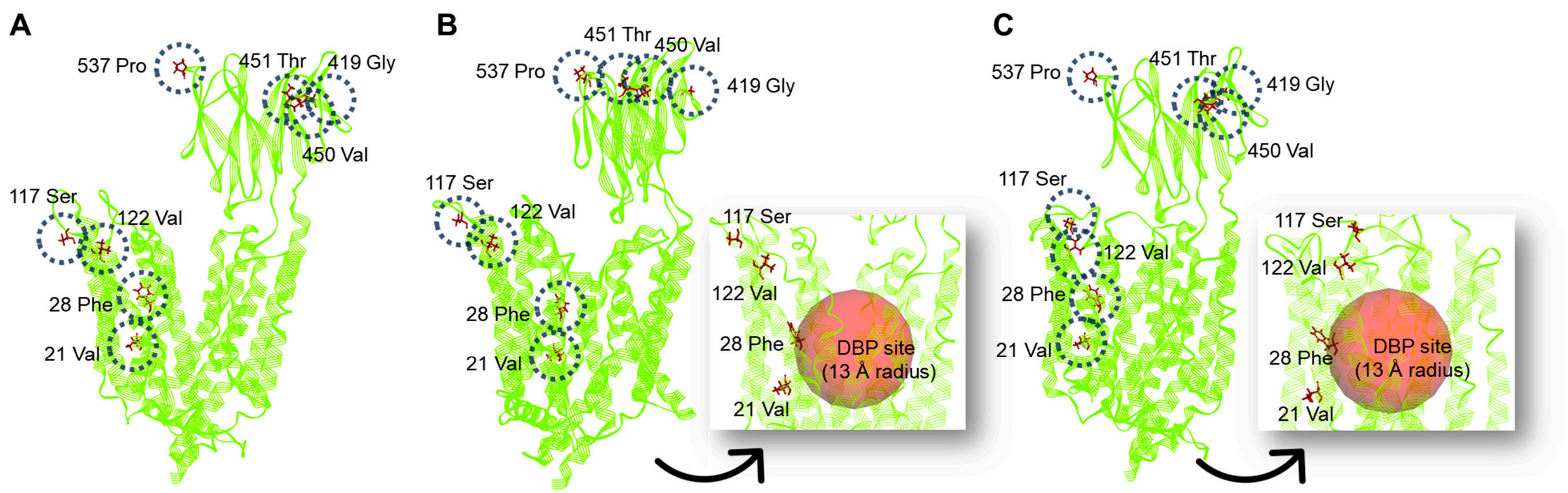

Figure S7. Location information of elements related to amino acid substitutions (as missense) in previously reported *SCL15A1* single nucleotide polymorphisms (SNP) in three conformations (apo-state [A], open conformation [B], and occluded conformation [C]) of PEPT1 outward facing. Proteins are presented in light green colored line ribbon display style. Blue dotted lines in the structure indicate amino acid substitution sites in each conformation. Amino acid regions of targeted point mutations are indicated with red colored stick type atoms. Curved arrows show enlarged images of drug binding pocket (DBP; indicated by a red sphere) and surrounding amino acids in PEPT1 outward facing open (B) and occluded (C) conformations.

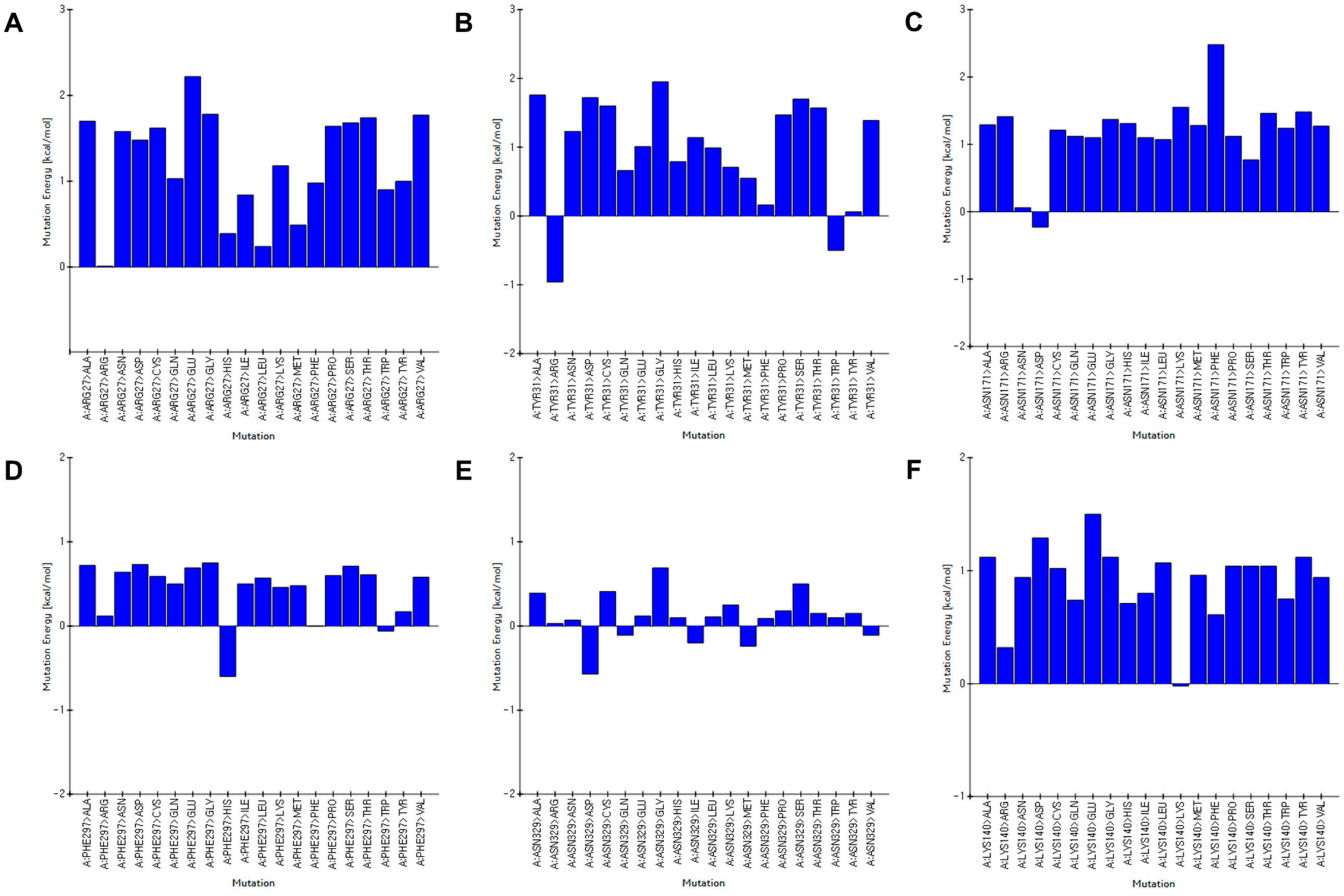
A
Mutation Energy [kcal/mol]
3
2
1
0
A:ARG27>ALA A:ARG27>ARG A:ARG27>ASN A:ARG27>ASP A:ARG27>CYS A:ARG27>GLN A:ARG27>GLU A:ARG27>GLY A:ARG27>HIS A:ARG27>ILE A:ARG27>LEU A:ARG27>LYS A:ARG27>MET A:ARG27>PHE A:ARG27>PRO A:ARG27>SER A:ARG27>THR A:ARG27>TRP A:ARG27>TYR A:ARG27>VAL
Mutation
B
Mutation Energy [kcal/mol]
3
2
1
0
-1
-2
A:TYR31>ALA A:TYR31>ARG A:TYR31>ASN A:TYR31>ASP A:TYR31>CYS A:TYR31>GLN A:TYR31>GLU A:TYR31>GLY A:TYR31>HIS A:TYR31>ILE A:TYR31>LEU A:TYR31>LYS A:TYR31>MET A:TYR31>PHE A:TYR31>PRO A:TYR31>SER A:TYR31>THR A:TYR31>TRP A:TYR31>TYR A:TYR31>VAL
Mutation
C
Mutation Energy [kcal/mol]
3
2
1
0
-1
-2
A:ASN171>ALA A:ASN171>ARG A:ASN171>ASN A:ASN171>ASP A:ASN171>CYS A:ASN171>GLN A:ASN171>GLU A:ASN171>GLY A:ASN171>HIS A:ASN171>ILE A:ASN171>LEU A:ASN171>LYS A:ASN171>MET A:ASN171>PHE A:ASN171>PRO A:ASN171>SER A:ASN171>THR A:ASN171>TRP A:ASN171>TYR A:ASN171>VAL
Mutation
D
Mutation Energy [kcal/mol]
2
1
0
-1
-2
A:PHE297>ALA A:PHE297>ARG A:PHE297>ASN A:PHE297>ASP A:PHE297>CYS A:PHE297>GLN A:PHE297>GLU A:PHE297>GLY A:PHE297>HIS A:PHE297>ILE A:PHE297>LEU A:PHE297>LYS A:PHE297>MET A:PHE297>PHE A:PHE297>PRO A:PHE297>SER A:PHE297>THR A:PHE297>TRP A:PHE297>TYR A:PHE297>VAL
Mutation
E
Mutation Energy [kcal/mol]
2
1
0
-1
-2
A:ASN329>ALA A:ASN329>ARG A:ASN329>ASN A:ASN329>ASP A:ASN329>CYS A:ASN329>GLN A:ASN329>GLU A:ASN329>GLY A:ASN329>HIS A:ASN329>ILE A:ASN329>LEU A:ASN329>LYS A:ASN329>MET A:ASN329>PHE A:ASN329>PRO A:ASN329>SER A:ASN329>THR A:ASN329>TRP A:ASN329>TYR A:ASN329>VAL
Mutation
F
Mutation Energy [kcal/mol]
2
1
0
-1
A:LYS140>ALA A:LYS140>ARG A:LYS140>ASN A:LYS140>ASP A:LYS140>CYS A:LYS140>GLN A:LYS140>GLU A:LYS140>GLY A:LYS140>HIS A:LYS140>ILE A:LYS140>LEU A:LYS140>LYS A:LYS140>MET A:LYS140>PHE A:LYS140>PRO A:LYS140>SER A:LYS140>THR A:LYS140>TRP A:LYS140>TYR A:LYS140>VAL
Mutation

G

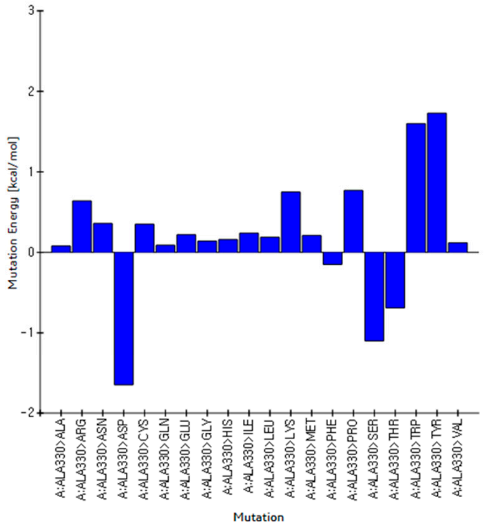

Figure S8. Structural binding (with cefaclor) energy profile results (at pH 7.4) according to application (sequentially for 20 amino acids) of point mutations of key interaction residues (27 Arg [A], 31 Tyr [B], 171 Asn [C], 297 Phe [D], 329 Asn [E], 140 Lys [F], and 330 Ala [G]) between PEPT1 and cefaclor explored in this study in PEPT1 outward facing open conformation.

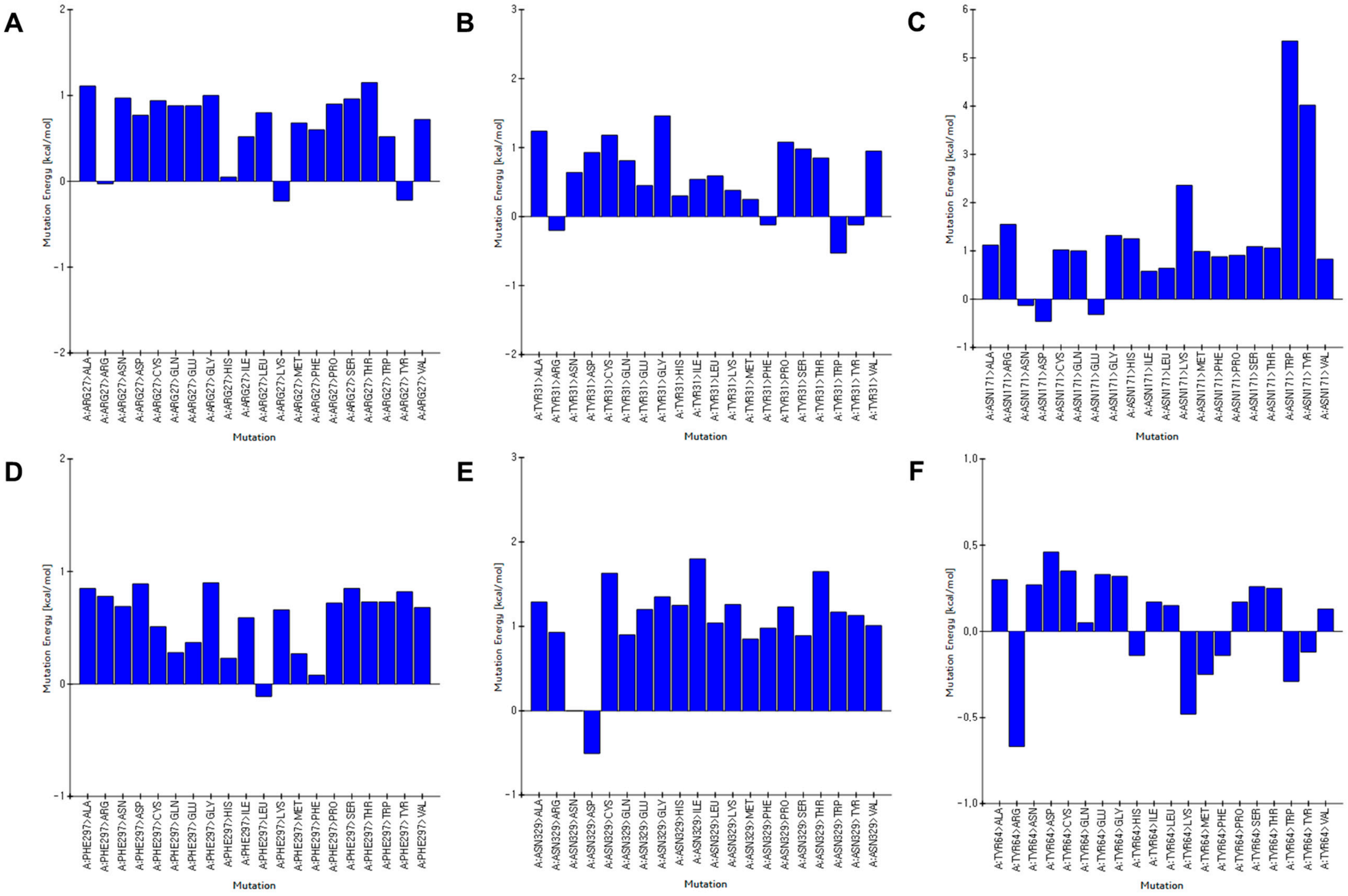
A
Mutation Energy [kcal/mol]
Mutation
A:ARG27>ALA
A:ARG27>ARG
A:ARG27>ASN
A:ARG27>ASP
A:ARG27>CYS
A:ARG27>GLN
A:ARG27>GLU
A:ARG27>GLY
A:ARG27>HIS
A:ARG27>ILE
A:ARG27>LEU
A:ARG27>LYS
A:ARG27>MET
A:ARG27>PHE
A:ARG27>PRO
A:ARG27>SER
A:ARG27>THR
A:ARG27>TRP
A:ARG27>TYR
A:ARG27>VAL
B
Mutation Energy [kcal/mol]
Mutation
A:TYR31>ALA
A:TYR31>ARG
A:TYR31>ASN
A:TYR31>ASP
A:TYR31>CYS
A:TYR31>GLN
A:TYR31>GLU
A:TYR31>GLY
A:TYR31>HIS
A:TYR31>ILE
A:TYR31>LEU
A:TYR31>LYS
A:TYR31>MET
A:TYR31>PHE
A:TYR31>PRO
A:TYR31>SER
A:TYR31>THR
A:TYR31>TRP
A:TYR31>TYR
A:TYR31>VAL
C
Mutation Energy [kcal/mol]
Mutation
A:ASN171>ALA
A:ASN171>ARG
A:ASN171>ASN
A:ASN171>ASP
A:ASN171>CYS
A:ASN171>GLN
A:ASN171>GLU
A:ASN171>GLY
A:ASN171>HIS
A:ASN171>ILE
A:ASN171>LEU
A:ASN171>LYS
A:ASN171>MET
A:ASN171>PHE
A:ASN171>PRO
A:ASN171>SER
A:ASN171>THR
A:ASN171>TRP
A:ASN171>TYR
A:ASN171>VAL
D
Mutation Energy [kcal/mol]
Mutation
A:PHE297>ALA
A:PHE297>ARG
A:PHE297>ASN
A:PHE297>ASP
A:PHE297>CYS
A:PHE297>GLN
A:PHE297>GLU
A:PHE297>GLY
A:PHE297>HIS
A:PHE297>ILE
A:PHE297>LEU
A:PHE297>LYS
A:PHE297>MET
A:PHE297>PHE
A:PHE297>PRO
A:PHE297>SER
A:PHE297>THR
A:PHE297>TRP
A:PHE297>TYR
A:PHE297>VAL
E
Mutation Energy [kcal/mol]
Mutation
A:ASN329>ALA
A:ASN329>ARG
A:ASN329>ASN
A:ASN329>ASP
A:ASN329>CYS
A:ASN329>GLN
A:ASN329>GLU
A:ASN329>GLY
A:ASN329>HIS
A:ASN329>ILE
A:ASN329>LEU
A:ASN329>LYS
A:ASN329>MET
A:ASN329>PHE
A:ASN329>PRO
A:ASN329>SER
A:ASN329>THR
A:ASN329>TRP
A:ASN329>TYR
A:ASN329>VAL
F
Mutation Energy [kcal/mol]
Mutation
A:TYR64>ALA
A:TYR64>ARG
A:TYR64>ASN
A:TYR64>ASP
A:TYR64>CYS
A:TYR64>GLN
A:TYR64>GLU
A:TYR64>GLY
A:TYR64>HIS
A:TYR64>ILE
A:TYR64>LEU
A:TYR64>LYS
A:TYR64>MET
A:TYR64>PHE
A:TYR64>PRO
A:TYR64>SER
A:TYR64>THR
A:TYR64>TRP
A:TYR64>TYR
A:TYR64>VAL

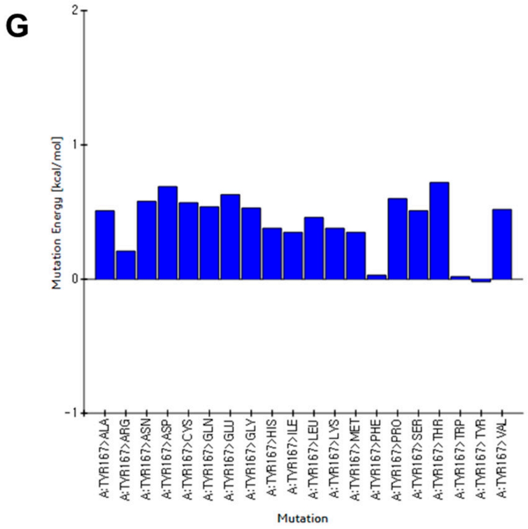

Figure S9. Structural binding (with cefaclor) energy profile results (at pH 7.4) according to application (sequentially for 20 amino acids) of point mutations of key interaction residues (27 Arg [A], 31 Tyr [B], 171 Asn [C], 297 Phe [D], 329 Asn [E], 64 Tyr [F], and 167 Tyr [G]) between PEPT1 and cefaclor explored in this study in PEPT1 outward facing occluded conformation.

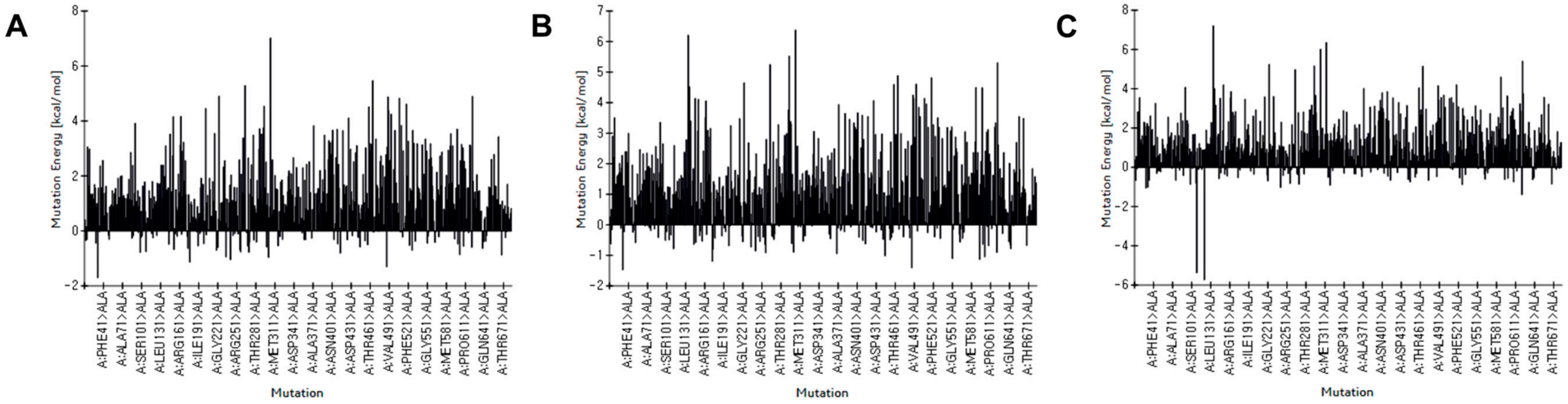

Figure S10. Structural stability analysis profile through alanine mutagenesis in three states (apo-state [A], open conformation [B], and occluded conformation [C]) of PEPT1 outward facing.

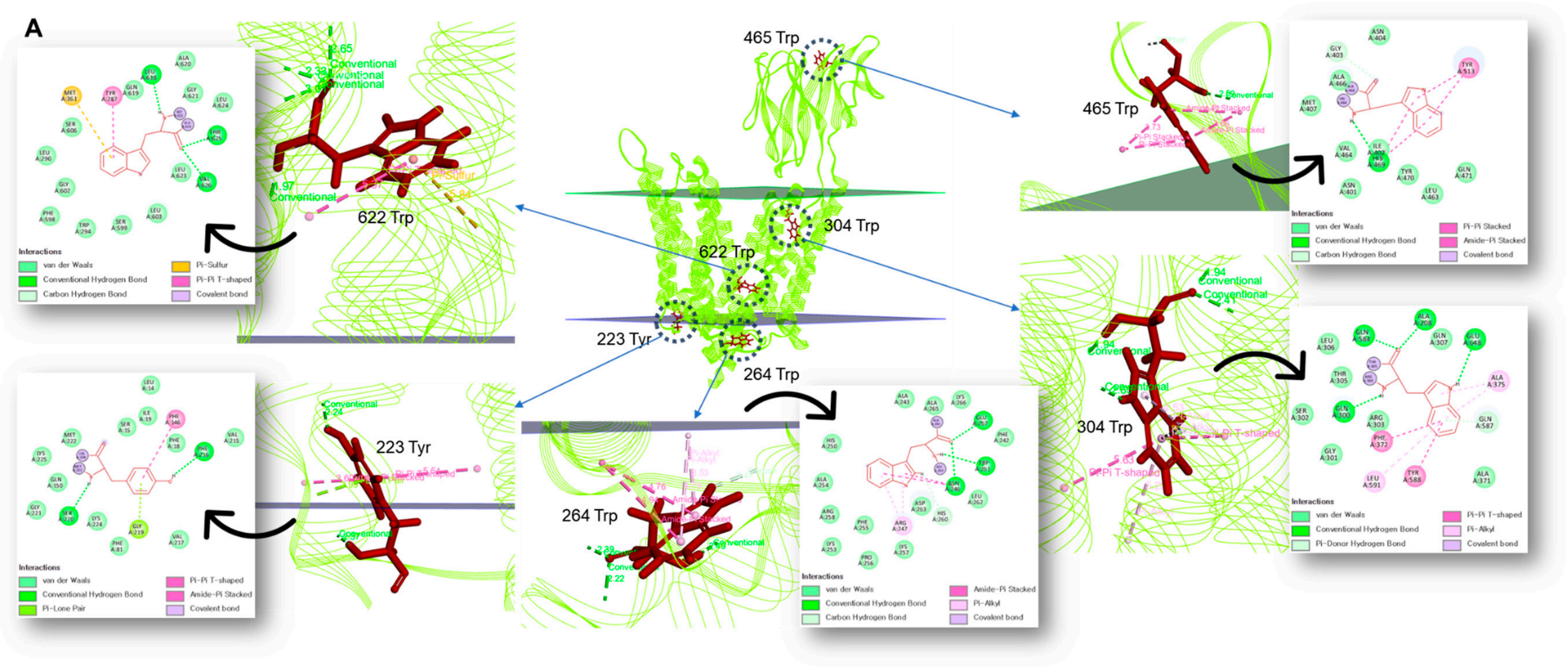

Figure S11 (continued)

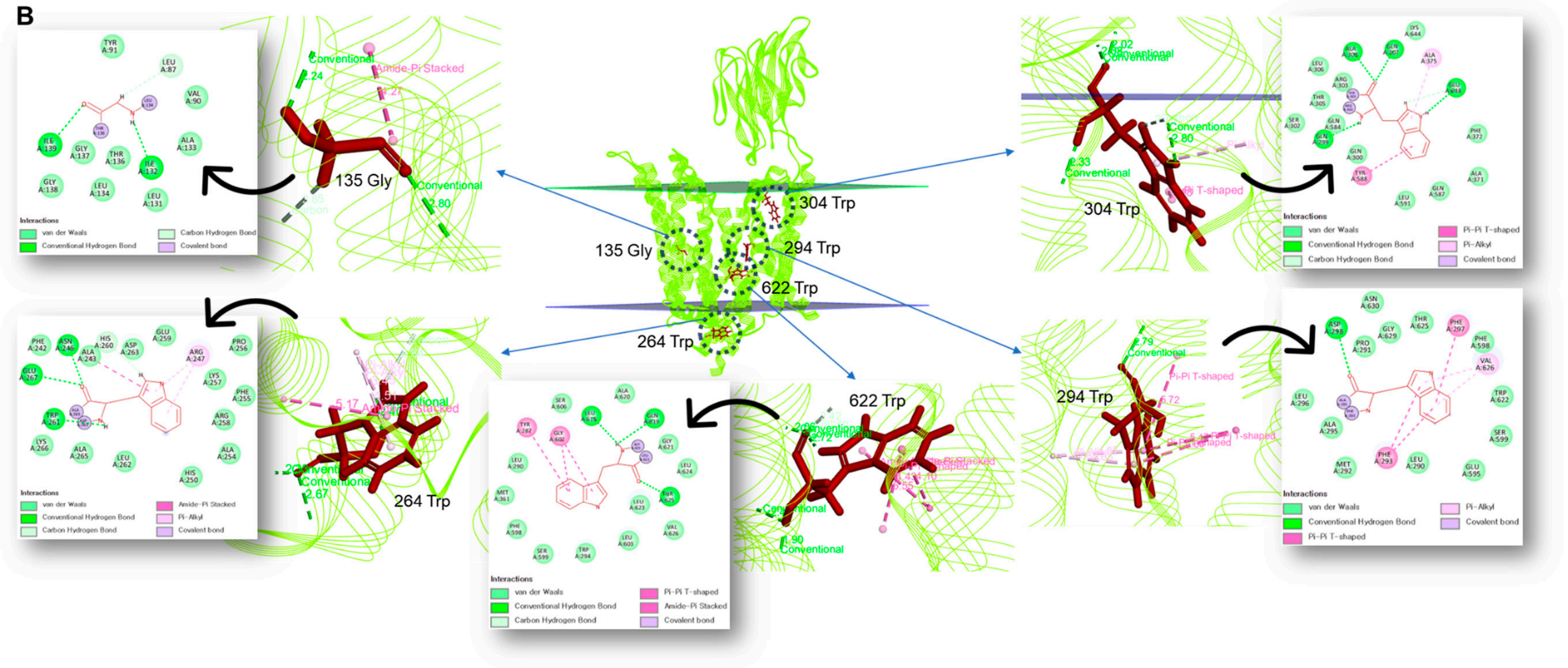

Figure S11 (continued)

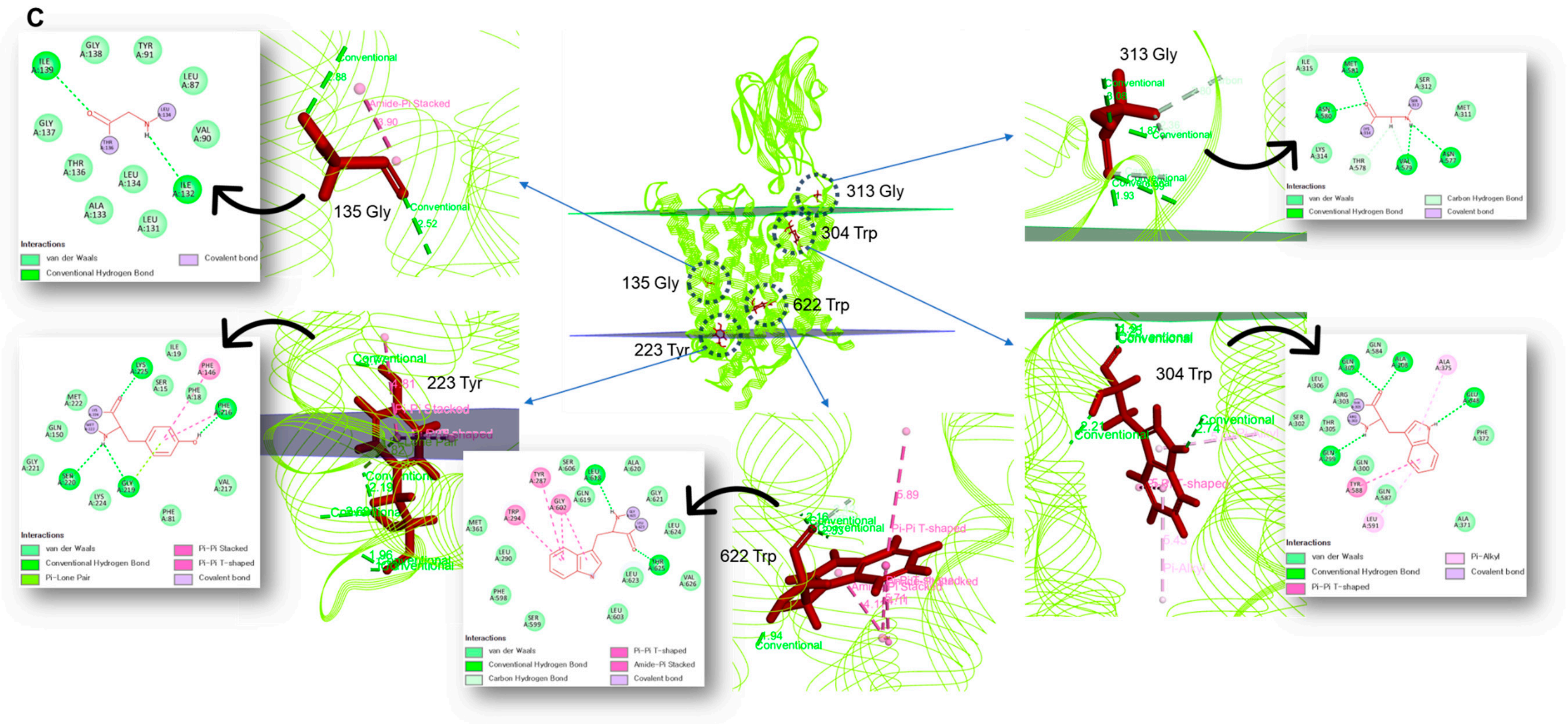

Figure S11. Analysis of amino acid positions (blue circle dotted lines) and non-bond interactions (colored dotted lines with length and type) within the PEPT1 structure for the top 5 structural instability derived after performing alanine mutagenesis in three states (apo-state [A], open conformation [B], and occluded conformation [C]) of PEPT1 outward facing. Proteins are presented in light green colored line ribbon display style. The top five amino acids causing structural instability discovered through alanine mutagenesis are indicated by red colored stick type atoms, and curved arrows show the two-dimensional interaction diagram within the structure of the corresponding amino acid. The blue straight lines in the figures represent enlargements of the corresponding regions in the PEPT1 structure. Green and blue planes in PEPT1 structures represent extracellular and cytosolic membranes, respectively.

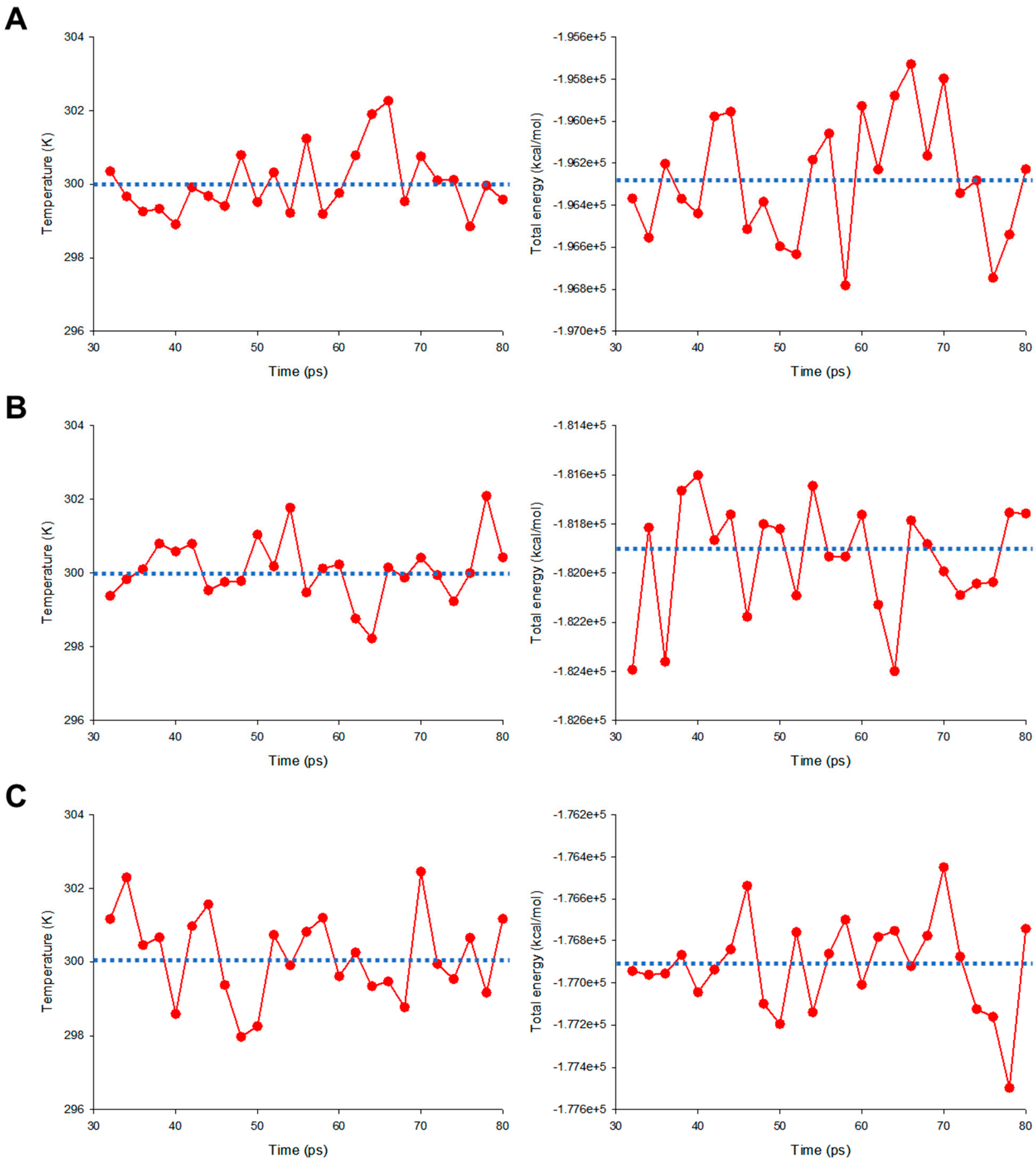

Figure S12 (continued)

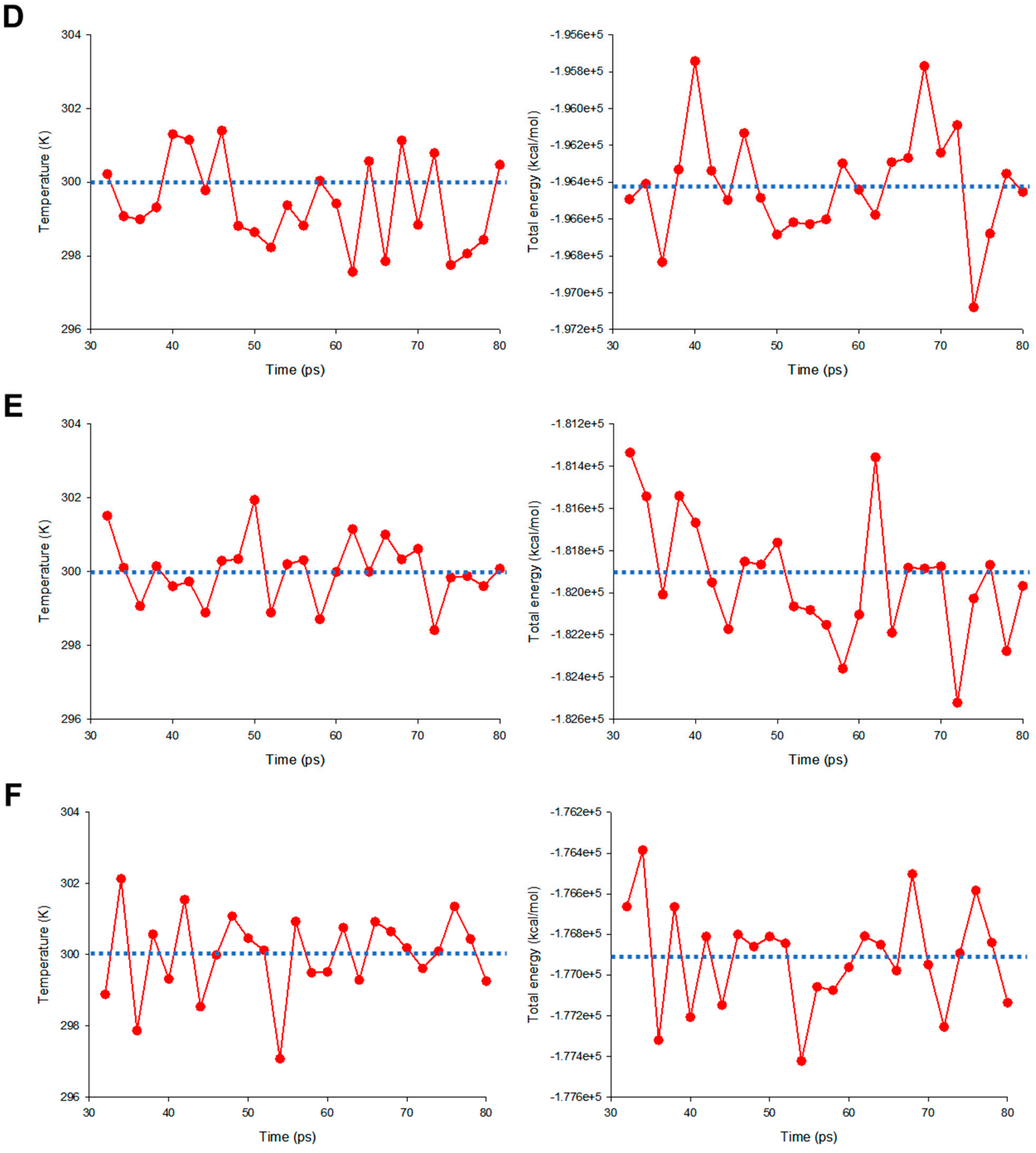

Figure S12. Results of thermodynamic structural stability during molecular dynamics simulations in PEPT1 outward facing apo-state (A and D), open conformation (B and E), and occluded conformations (C and F) according to genetic polymorphisms of *SLC15A1* exon 5 (c.381G>A; A-C) or 16 (c.1287G>C; D-F). The left and right graphs in A-F show temperature and structural energy profiles over time at the final production stage in molecular dynamics simulation, respectively. The blue horizontal dotted lines in the graph represent the average values of the temperature and energy changes of the protein within the simulation time.
